# A novel super-resolution microscopy platform for cutaneous alpha-synuclein detection in Parkinson’s disease

**DOI:** 10.3389/fnmol.2024.1431549

**Published:** 2024-09-04

**Authors:** Ofir Sade, Daphna Fischel, Noa Barak-Broner, Shir Halevi, Irit Gottfried, Dana Bar-On, Stefan Sachs, Anat Mirelman, Avner Thaler, Aviv Gour, Meir Kestenbaum, Mali Gana Weisz, Saar Anis, Claudio Soto, Melanie Shanie Roitman, Shimon Shahar, Kathrin Doppler, Markus Sauer, Nir Giladi, Nirit Lev, Roy N. Alcalay, Sharon Hassin-Baer, Uri Ashery

**Affiliations:** ^1^School of Neurobiology, Biochemistry, Biophysics, Life Sciences Faculty, Tel Aviv University, Tel Aviv, Israel; ^2^Sagol School of Neuroscience, Tel Aviv University, Tel Aviv, Israel; ^3^Department of Biotechnology and Biophysics, Biocenter, University of Würzburg, Würzburg, Germany; ^4^Movement Disorders Division, Neurological Institute, Tel Aviv Sourasky Medical Center, Tel Aviv, Israel; ^5^Faculty of Medical and Health Sciences, Tel Aviv University, Tel Aviv, Israel; ^6^Department of Neurology, Meir Medical Center, Kfar Saba, Israel; ^7^Department of Neurology, Movement Disorders Institute, Chaim Sheba Medical Center, Ramat Gan, Israel; ^8^Mitchell Center for Alzheimer’s Disease and Related Brain Disorders, University of Texas Medical School, Houston, TX, United States; ^9^Department of Statistics, Exact Sciences Faculty, Tel Aviv University, Tel Aviv, Israel; ^10^Department of Neurology, University Hospital Würzburg, Würzburg, Germany

**Keywords:** alpha-synuclein aggregates, biomarker, density-based spatial clustering of applications with noise (DBSCAN), direct stochastic optical reconstruction microscopy (*d*STORM), early diagnosis, fast optimized cluster algorithm for localizations (FOCAL), Parkinson’s disease, super-resolution microscopy

## Abstract

Alpha-synuclein (aSyn) aggregates in the central nervous system are the main pathological hallmark of Parkinson’s disease (PD). ASyn aggregates have also been detected in many peripheral tissues, including the skin, thus providing a novel and accessible target tissue for the detection of PD pathology. Still, a well-established validated quantitative biomarker for early diagnosis of PD that also allows for tracking of disease progression remains lacking. The main goal of this research was to characterize aSyn aggregates in skin biopsies as a comparative and quantitative measure for PD pathology. Using direct stochastic optical reconstruction microscopy (*d*STORM) and computational tools, we imaged total and phosphorylated-aSyn at the single molecule level in sweat glands and nerve bundles of skin biopsies from healthy controls (HCs) and PD patients. We developed a user-friendly analysis platform that offers a comprehensive toolkit for researchers that combines analysis algorithms and applies a series of cluster analysis algorithms (i.e., DBSCAN and FOCAL) onto *d*STORM images. Using this platform, we found a significant decrease in the ratio of the numbers of neuronal marker molecules to phosphorylated-aSyn molecules, suggesting the existence of damaged nerve cells in fibers highly enriched with phosphorylated-aSyn molecules. Furthermore, our analysis found a higher number of aSyn aggregates in PD subjects than in HC subjects, with differences in aggregate size, density, and number of molecules per aggregate. On average, aSyn aggregate radii ranged between 40 and 200 nm and presented an average density of 0.001–0.1 molecules/nm^2^. Our *d*STORM analysis thus highlights the potential of our platform for identifying quantitative characteristics of aSyn distribution in skin biopsies not previously described for PD patients while offering valuable insight into PD pathology by elucidating patient aSyn aggregation status.

## Introduction

Parkinson’s Disease (PD) is the second most prevalent neurodegenerative disease after Alzheimer’s disease (AD). PD affects 0.1–0.2% of the general population and 1% of individuals over the age of 60 worldwide (Tysnes and Storstein, 2017). PD is diagnosed clinically based on the presence of bradykinesia and either resting tremor or rigidity (Massano and Bhatia, 2012; Poewe et al., 2017; Berg et al., 2018). Most of the tests, however, fail to provide a quantitative, non-biased assessment of patient status. Although PD starts to develop years before its clinical diagnosis, early diagnosis before and during the prodromal state of the overt clinical symptoms remains an unmet need. Yet, with tests such as DaT SPECT, F-dopa PET, seeding amplification assays, sleep studies, and smell identification tests, preclinical diagnosis may become feasible in the near future. Recently, a new biologic diagnostic strategy based on neuronal alpha-Synuclein (aSyn) pathology has been proposed for the biological staging of PD (Simuni et al., 2024).

aSyn is a synaptic protein enriched in nerve terminals that regulates synaptic transmission (Burré et al., 2014), and is found physiologically both as a monomer and a tetramer (Nuber et al., 2024). Under pathological conditions, natively unfolded aSyn molecules can self-aggregate into pathological oligomers. These structures can then extend into β -sheet-rich amyloid protofibrils and fibrils and more complex formations, such as soluble fibrils or ribbons, which deposit into Lewy bodies (LBs) and Lewy neurites that accumulate in the brain neurons (Stefanis, 2012; Brás and Outeiro, 2021). Although the particular aSyn conformation responsible for the neurotoxicity of pathogenic aSyn has yet to be determined, it is presumed that small soluble oligomers lead to toxicity (Conway et al., 2000; Goldberg and Lansbury, 2000; Lashuel et al., 2013; Santos et al., 2023; Chen et al., 2024). While brain aSyn aggregation and the presence of LBs are hallmarks of PD, aSyn aggregates in peripheral tissues outside the central nervous system have also been demonstrated. These include the enteric nervous system of the gastrointestinal tract, which might be associated with *gut-first* PD (Videlock et al., 2023), sub-mandibular glands, skin, and other peripheral organs (Almikhlafi, 2024). This led to the notion that aSyn aggregates can be detected in peripheral tissues, even years before the appearance of PD motor symptoms. Accordingly, biosamples from the skin, the gastrointestinal tract, sub-mandibular glands, cerebrospinal fluid (CSF), and saliva have been extensively examined (Shannon et al., 2012; Mollenhauer et al., 2013; Donadio et al., 2014; Doppler et al., 2014; Adler et al., 2016; Goldman et al., 2018; Vivacqua et al., 2019). However, these studies reported diverging findings regarding the sensitivity and specificity of aSyn as a biomarker for PD (Lebouvier et al., 2008; Shannon et al., 2012; Tsukita et al., 2019).

Biopsies from more accessible tissues, such as the skin, which contains sensory and autonomic nerves that intricately innervate sweat glands, blood vessels, and pilomotor muscles, have been addressed (Adler et al., 2016). In most such studies, immunohistochemical assays were performed to assess the levels of native aSyn and phosphorylated (p)-aSyn deposits, corresponding to the pathological form of aSyn (Okochi et al., 2000), in dermal autonomic nerve fibers to distinguish between PD patients and Healthy control (HC) subjects (Wang et al., 2013; Donadio et al., 2014, 2019; Gibbons et al., 2016, 2021; Antelmi et al., 2017; Doppler et al., 2017; Beach et al., 2018; Tsukita et al., 2019; Giannoccaro et al., 2020; Doppler, 2021). p-aSyn deposits were observed in the dermal autonomic innervation of blood vessels, erector pili muscles, and sweat glands and were suggested to be associated with autonomic neurodegeneration in PD patients (Donadio et al., 2014, 2019, 2024; Doppler et al., 2014; Antelmi et al., 2017; Gibbons et al., 2024). Recently, novel seeding assays, such as real-time quaking-induced conversion (RT-QuIC) and protein misfolding cyclic amplification (PMCA), now referred to as the aSyn seeding amplification assay (SAA), were used to detect aSyn aggregation in skin tissue homogenates (Manne et al., 2020). These studies showed promising results regarding the ability to differentiate between PD patients and HC samples but have yet to meet the requirements needed to become clinically adopted.

One of the main limitations of conventional immunoassays is their inability to resolve small aggregates that are below the resolution of conventional light microscopy (200–250 nm) (Huang et al., 2009; Sigrist and Sabatini, 2012; Sauer and Heilemann, 2017; Sahl and Hell, 2019; Dankovich and Rizzoli, 2021), and, thus, studies based on such approaches mostly overlooked the composition, dispersion, density, and size of aSyn aggregates. Similarly, aSyn-SAAs do not provide information on the initial aggregate seed status. In recent years, the application of super-resolution microscopy (SRM) to detect protein aggregates in neurodegenerative diseases has provided novel information about the sizes, shapes, and densities of the presumably toxic small-to medium-sized aggregates implicated in the pathology of such conditions. Using an SRM method, namely, *d*STORM, allowed for the quantification and characterization of aSyn aggregates in brain slices of a transgenic PD mouse model (Wegrzynowicz et al., 2019). The aggregates detected were classified into different patient populations that correlated with the degree of PD-like symptoms. Most of the aSyn aggregates, which were associated with disease progression, were 20–250 nm in size, which is below the diffraction limit of conventional microscopes. The use of SRM also revealed that the aSyn aggregate modifier anle138b was able to specifically reduce the levels of medium-sized aSyn aggregates, which was correlated with improvement in mouse motor function (Wegrzynowicz et al., 2019). Indeed, SRM has been used to characterize amyloid-beta (Aβ) and tau aggregates in CSF samples of AD patients and allowed for distinguishing AD patients from HC subjects (Zhang et al., 2015). Together, these results provide proof of concept for the use of SRM, specifically *d*STORM, to detect sub-diffraction-sized aSyn aggregates, allowing such particles to serve as an accessible biomarker of PD.

Here, we present a novel combination of SRM and an advanced analysis platform, which improves imaging and detection sensitivity. When applied to biological samples from PD patients and HC subjects, this combination enabled the detailed characterization of nano-sized aSyn aggregates and their classification according to different quantitative parameters, allowing the development of a new molecular biomarker for PD.

## Materials and methods

### Biopsy preparation and processing

Participants were recruited from three medical centers in Israel: Tel Aviv Sourasky Medical Center, Meir Medical Center, and Sheba Medical Center. The study was approved by the hospitals’ medical ethics committees (ethics committee approvals 8492–21, 0257–08, and 0302–23, respectively). All subjects gave written informed consent.

**Figure 1:**
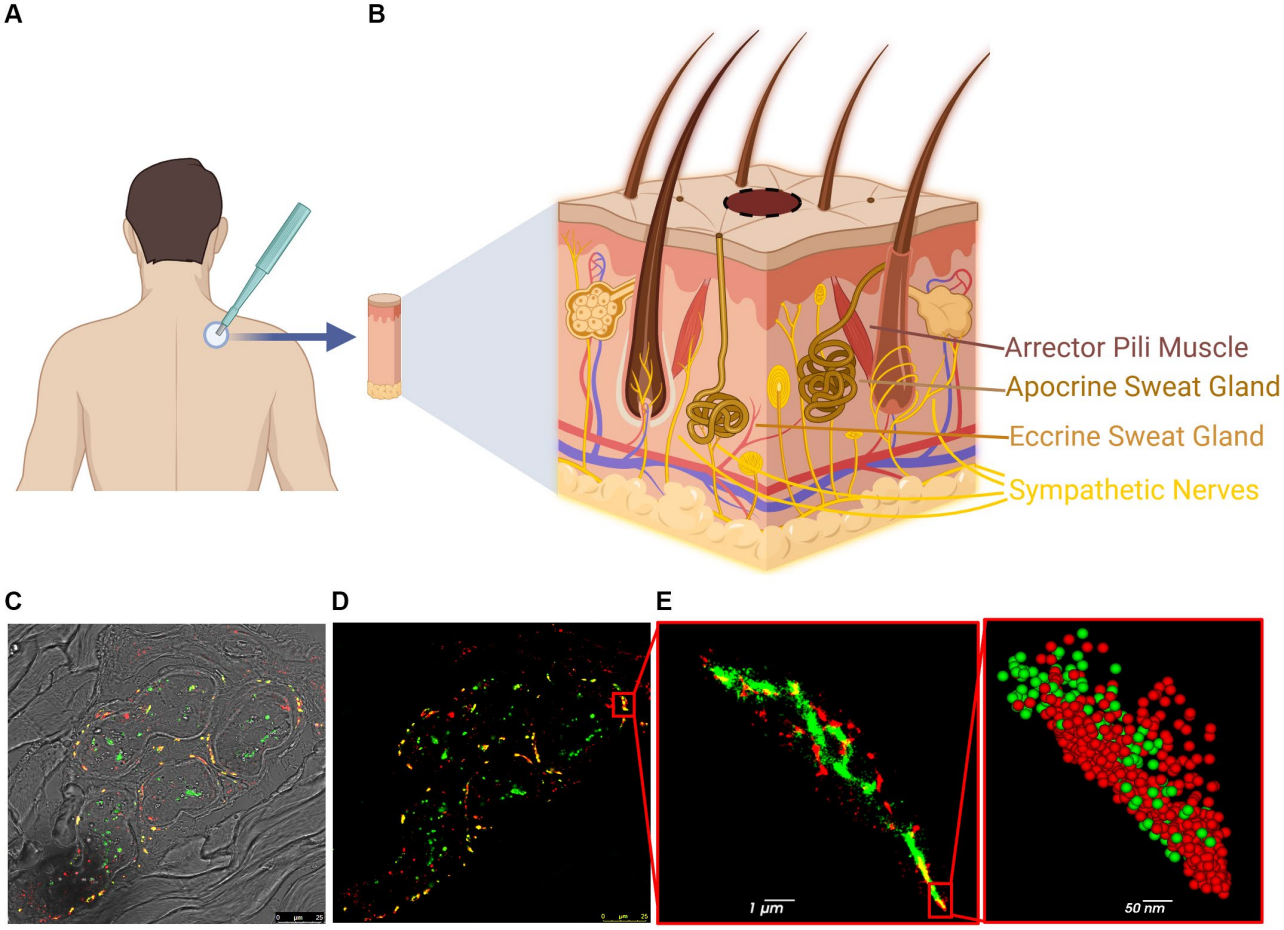
Steps and methodology of skin biopsy processing for *d*STORM. **(A)** Skin biopsy taken from the upper back (C7). **(B)** The skin contains various innervated structures, such as erector pili muscles, sweat glands, autonomic nerves, and cutaneous nerves. **(C,D)** Confocal image of a sweat gland from PD patient: p-aSyn (red), neuronal marker PGP9.5 (green) and areas of co-localization (yellow). **(E)** *d*STORM reconstructed image of part of the sweat gland innervation (insert in **D**) shows p-aSyn (red) and PGP9.5 (green) and their co-localization (yellow). Insert-a close-up view showing single-molecule distribution of both p-aSyn (red) and PGP9.5 (green).

A single skin biopsy from the upper back (at level C7) of each PD patient (seven total: five males and two females) and each HC subject (seven total: five males and two females) was obtained by clinicians at the three medical centers using a 3–4 mm punch (Figure 1A and Supplementary Table S1). All subjects were clinically evaluated by a movement disorders neurologist using the Movement Disorder Society-Unified Parkinson’s Disease Rating Scale (MDS-UPDRS), Montreal Cognitive Assessment (MoCA), and Hoehn and Yahr (H&Y) scales to assess PD severity (mean UPDRS: 34. mean MoCA: 23.7. mean H&Y: 2.2) and to confirm that the HC subjects were free of disease (mean UPDRS: 0.86. Mean MoCA: 26.6. All H&Y: 0). The biopsies were fixed in 4% paraformaldehyde (PFA) overnight, moved to 15% sucrose solution for 1 h and then to a 30% sucrose solution overnight. The samples were frozen in optimal cutting temperature (OCT) compound and kept at −80°C until used. The samples were then sliced using a cryostat (Leica CM3050-S) into 12 μm-thick sections and mounted on 1.5H positively charged coverslips or glass slides.

For subsequent analysis, biopsies from 7 HC and 7 PD samples (Supplementary Table S1) were immunostained and analyzed (Figure 1). Skin sections were stained with an anti-p-aSyn antibody (mouse anti-phosphorylated-aSyn antibody (Ser129), diluted 1:500; 825,701, Biolegend). Subsequent skin sections from the same 4 HC subjects and 4 PD patients were stained with an anti-total aSyn (t-aSyn) antibody (mouse anti-α-synuclein antibody, clone 42, 1:250 dilution; 610,787, BD Bioscience). Both conditions were also stained with anti-protein gene product 9.5 (PGP9.5), a neuronal marker (rabbit anti-PGP9.5 antibody, diluted 1:200; ab108986, Abcam) (Supplementary Table S1) and nerve areas that showed PGP9.5 staining were imaged with an anti-aSyn antibody as detailed above. Samples from one PD patient and one HC were excluded from analysis due to tissue disintegration, leaving six PD patients (four males and two females; median age: 69.5) and six HC subjects (five males and one female; median age: 65.5) for the analysis. For each subject, 10 images from 2 to 3 slices were analyzed.

### Confocal microscopy

All skin sections from each subject were initially imaged with a confocal scanning laser microscope (Leica SP8). Images (1,024 × 1,024 pixels) were acquired using an HC PL APO CS2 63X/1.40 NA oil objective, DD 488/552 and 638 excitation beam splitters, and optically pumped semi-conductor lasers (OPSLs) at 552 and 638 nm lasers (Leica Microsystems). Images were obtained by averaging four scans using LasX software (Leica Microsystems). This step was performed to confirm antibody staining and the presence of a sweat gland or nerve bundle.

### Super-resolution microscopy

*d*STORM was performed using a single-molecule localization microscope (Vutara350, Bruker) (Wegrzynowicz et al., 2019; Sade et al., 2023; Sela et al., 2023). Briefly, in *d*STORM, fluorophores are photo-switched between bright and dark states in a stochastic manner (Supplementary Figure S1), with the position of a single fluorophore (i.e., molecule) being estimated to a resolution of ~10–20 nm by fitting a point spread function (PSF). The sample is imaged for 3,000 frames. In each frame, stochastic activation of different molecules occurs, allowing for the separation of closely positioned molecules and subsequent reconstruction of an inclusive image of all localizations in the image (Supplementary Figures S1C,D). The improved resolution of *d*STORM (~10–20 nm), as compared to that of confocal microscopy (~200 nm), enables the detection of single molecules and populations of sub-diffractional aggregates, that would otherwise be indistinguishable.

A combination of two laser wavelengths was used (i.e., 561 and 647 nm), with laser power of 1,000 mW with a range of 5–10 kW/cm2 (for the 562 nm laser, we measured at the objective plane 116 mW and for the 647 nm laser 110 mW at 100%). In the scans introduced in this paper, 30% laser power was applied, reaching approximately 1.5–3 kW/cm2. The Vutara350 microscope is equipped with a bi-plane detection option, which allows three-dimensional imaging of up to a few hundred nanometers (SRX Full System Manual|SRX Manual, 2022). The Vutara350 microscope also uses a water immersion 60x objective, 1.20NA, with a field of view of 10 μm × 10 μm. The cameras used were a sCMOS camera (4 MP, 6.5 μm × 6.5 μm pixel size for super-resolution imaging) and a CCD camera (1,392 × 1,040 pixels, for wide-field imaging) with an exposure time of 20 ms per frame and 20 ms between frames. We acquired a total of 3,000 frames at each wavelength (i.e., 561 and 647 nm), or 6,000 frames in total, and fitted a PSF to each detected particle using VutaraSRX software. Particles with skewed or wide PSFs were discarded. High-quality PSFs were used to convert particles from a pixelated image into precise single-molecule localizations, represented by three-dimensional coordinates that correspond to the peak of the fitted PSF, using VutaraSRX software.

### Image analysis

Several modules were implemented to analyze *d*STORM output data. Each subject was represented by a vector consisting of one average value from each of their *d*STORM images (*n* = 30 for t-aSyn, *n* = 10 for p-aSyn). The statistical test used was Mann–Whitney *U* (MWU) test due to the small sample sizes. All the analysis was performed using self-written Python code, using open-source libraries.

We created a tailor-made platform for the cluster analysis of *d*STORM images programmed in Python using open-source libraries. The platform included three cluster-identification algorithms: density-based spatial clustering of applications with noise (DBSCAN) (Ester et al., 1996; Pedregosa et al., 2011a), hierarchical density-based spatial clustering of applications with noise (HDBSCAN) (Mcinnes et al., 2016; McInnes et al., 2017), and fast optimized cluster algorithm for localizations (FOCAL) (Mazouchi and Milstein, 2016; Nino et al., 2020). Considering the strengths and limitations of each algorithm allowed us to select the most suitable approach for a given dataset and analysis objective. We initially applied the DBSCAN and FOCAL algorithms to our datasets (Supplementary Figure S4) and found that, as described below, they most accurately characterized aSyn aggregates in skin biopsies. DBSCAN-based searches for areas of high density in the dataset and defines clusters. The Sci-Kit DBSCAN library (Python3.6) was used here (Pedregosa et al., 2011a). FOCAL examines the number of localizations per 3D voxel and defines minimal numbers of localizations per voxel (*minL*) and per neighboring voxels (*minC*). As *d*STORM output provides information in addition to localization coordinates, specifically the intensity or photon count (PC) at each localization, we implemented an adjustment to FOCAL (FOCAL^PC^) and defined a new parameter, namely, the minimum average photon count (*maPC*) in a group of voxels considered as a cluster candidate (Supplementary Figure S2). In this manner, we also give weight to the intensity at each localization, which correlated with localization precision (Supplementary Figure S3).

Various parameter combinations were tested using FOCAL to optimize skin p-aSyn cluster detection. All parameter combinations were supplemented with a PCA of one standard deviation for noise reduction, a minimum threshold of PC of 1,000, and a maximum threshold of x-precision of 100 nm. The average PC at all localizations in each voxel was calculated and only voxels with an average PC higher than the *maPC* were reconsidered as clusters. If the new voxel group met the *minL* and *minC*, it was considered a cluster; otherwise, it was discarded. This adjustment allowed FOCAL^PC^ to detect clusters containing more intense localizations, which we found to be correlated with higher localization precision (Supplementary Figure S3). This adjustment was further used to ensure that the clustering was strict and did not allow localizations that strongly deviated spatially from most localizations in the cluster. For convenience, density measures in the platform were multiplied by a factor of 1,000.

## Results

As *d*STORM data provide single-molecule resolution, one can estimate both the overall distribution of aSyn molecules in skin nerves and their organization into aggregates. Accordingly, we first performed a basic comparison of the number of aSyn molecules that were detected in all conditions and their characteristics (Figures 1A,B). We compared the number of localizations (estimated for the number of molecules detected), their localization precision, and their correlation with their intensity (measured as a PC). The number of localizations per image was normalized per image region of interest (ROI) volume to account for possible bias in ROI selection between PD patients and HC samples. The average number of localizations per *d*STORM image normalized to ROI volume was similar for t-aSyn in both sets of subjects (PD patients: 2.23e-07 ± 1.3e-08, *n* = 40 images; HC subjects: 2.48e-07 ± 8.4e-09, *n* = 40 images). The average number and distribution of the number localizations for p-aSyn were also similar (PD patients: 2.4e-07 ± 9.2e-09, *n* = 60; HC subjects: 2.4e-07 ± 1e-08, *n* = 60).

We next examined the precision of each localization by correlating the precision of each localization with its intensity (PC) and validated that for high PC values, the localization precision was better for both t-aSyn and p-aSyn in both subject populations (Supplementary Figure S3). We also found that the localization precision was similar for PD patients and HC subjects for both t-aSyn and p-aSyn in the X, Y, and Z axes, allowing for the use of the same clustering algorithms on all data sets. As localization precision was lower for localizations with PC values lower than 1,000, we defined a minimum PC of 1,000 as a cutoff for further analysis, as localizations with lower values showed lower precision. Together, these results suggest that overall, the number and parameter of each aSyn molecule in both PD patients and HC subjects are similar. This allowed us to continue onto the next stages of analysis and compare the distribution of aSyn molecules in PD patients and HC subjects.

### Less nerve fiber tissue is preserved in areas enriched with phosphorylated-aSyn

**Figure 2:**
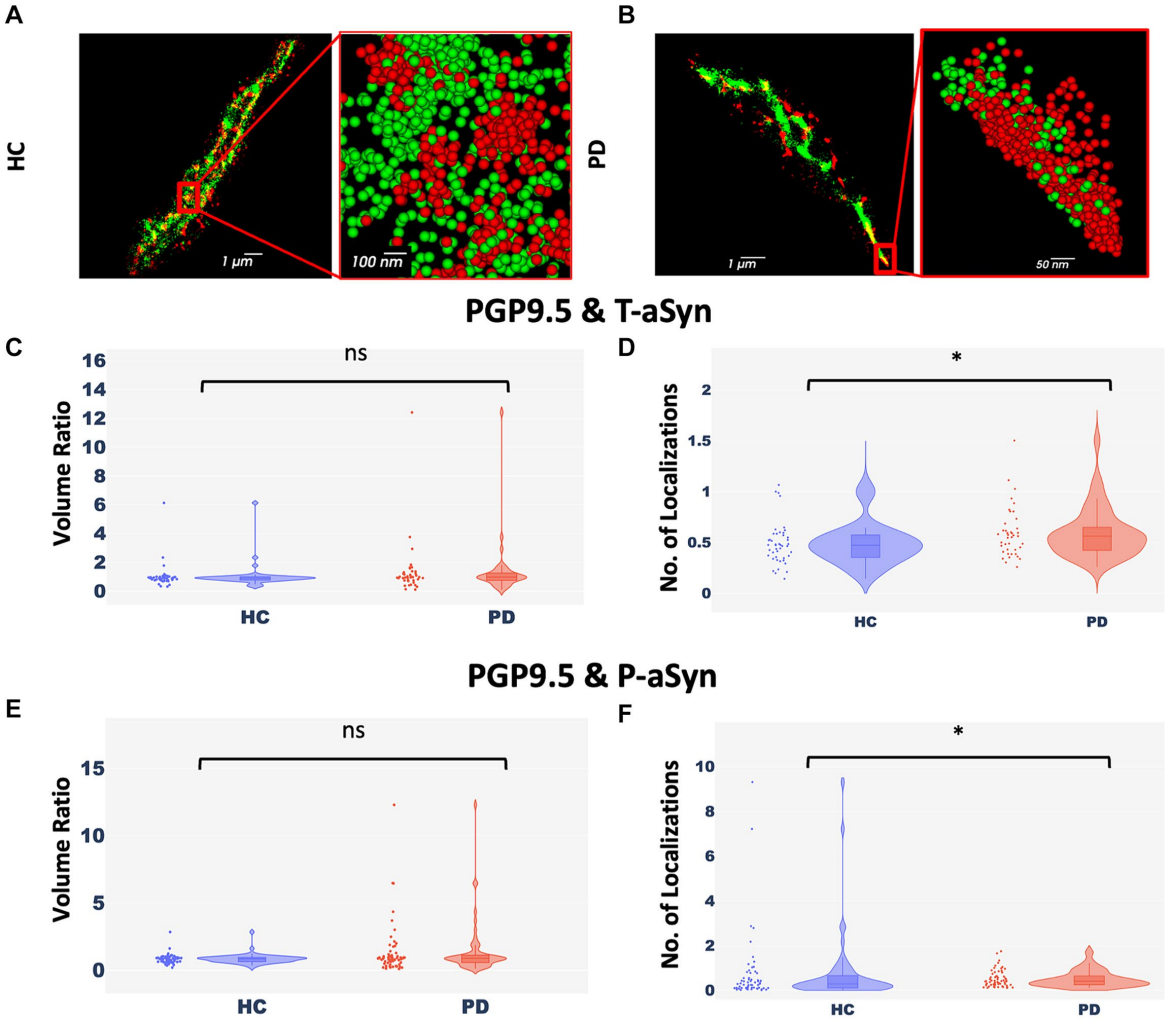
Nerve cells enriched with p-aSyn are less well-preserved. **(A,B)** Reconstructed *d*STORM images showing p-aSyn localization (red) and that of a neuronal marker (green) in sweat gland innervation in a PD patient **(A)** and an HC subject **(B)**. **(A)** and **(B)** insert - a close-up view showing single molecules distribution of both t-aSyn (red) and PGP9.5 (green). **(C)** The ratio of PGP9.5 ROI volume to t-aSyn ROI volume within each *d*STORM image is not significantly different between HC subjects (blue) [Median: 0.89±0.125, *n* = 40] and PD patients (red) [Median: 0.97±0.295, *n* = 40], with a *p*-value of 0.165. **(D)** The ratio of the number of PGP9.5 localizations to t-aSyn localizations is significantly different between HC subjects (blue) [Mean (median) – 0.485±0.03 (0.47)] and PD patients (red) [Mean (median) – 0.59±0.04 (0.56)], with a *p*-value of 0.026. **(E)** The ratio of PGP9.5 ROI volume to p-aSyn ROI volume within each *d*STORM image is not significantly different between HC subjects [Median: −18.5e+09±10.7e+09, *n* = 60] and PD patients [Median: −21.6e+09±5e+09, *n* = 60], with a *p*-value of 0.39. **(F)** The ratio of the number of PGP9.5 localizations to p-aSyn localizations is significantly higher in HC subjects (blue) [Mean (median) – 0.74±0.2 (0.29)] compared to PD patients (red) [Mean (median) – 0.51±0.05 (0.41)] (each point represents a ratio in a single *d*STORM image), with p-value of 0.03. *p < 0.05.

Peripheral nerve cells undergo degeneration in PD patients (Mu et al., 2013; Comi et al., 2014; Marogianni et al., 2020; Paul et al., 2020; Sharabi et al., 2021; Vacchi et al., 2021). To examine if there was a correlation between the appearance of p-aSyn molecules and degeneration of peripheral nerve fibers in the skin (Figures 2A,B), we compared two parameters, namely, the ratio of volumes covered by aSyn and by a neuronal marker (PGP9.5) (Figures 2C,E) and the ratio of the number of localizations of aSyn and a neuronal marker (PGP9.5) in these volumes (Figures 2D,F). No significant differences between subject groups were observed in ratios of the volume of both t-aSyn and p-aSyn to nerve fiber area in both HC subjects (Figure **2**C) and PD patients (Figure **2**E). However, we hypothesized that in nerve tissues, over-abundance of aSyn molecules and especially with p-aSyn molecules could cause damage to the nerve fiber or synapse of autonomic nerve fibers, such that less neuronal signal should be detected. Our results show that the number of t-aSyn molecules that were detected, as compared to a neuronal marker (PGP9.5), was significantly higher in PD subjects than HC subjects (Figure 2D). However, the number of p-aSyn molecules that were detected, as compared to a neuronal marker (PGP9.5), was significantly lower in PD patients as compared to HC subjects (Figure 2F). This suggests that in areas enriched with p-aSyn, less nerve fiber tissue is preserved. We next examined the areas marked by the neuronal marker (PGP9.5) over 200 × 200 μm regions of the sweat gland/nerve bundle in wide-field images containing the areas that were imaged in *d*STORM. We found that the neuronal marker labeling is significantly higher in HC subjects compared to PD patients (Supplementary Figure S5), as previously demonstrated (Mu et al., 2013; Comi et al., 2014; Marogianni et al., 2020; Paul et al., 2020; Sharabi et al., 2021; Vacchi et al., 2021).

### PD patients present more p-aSyn aggregates

**Figure 3:**
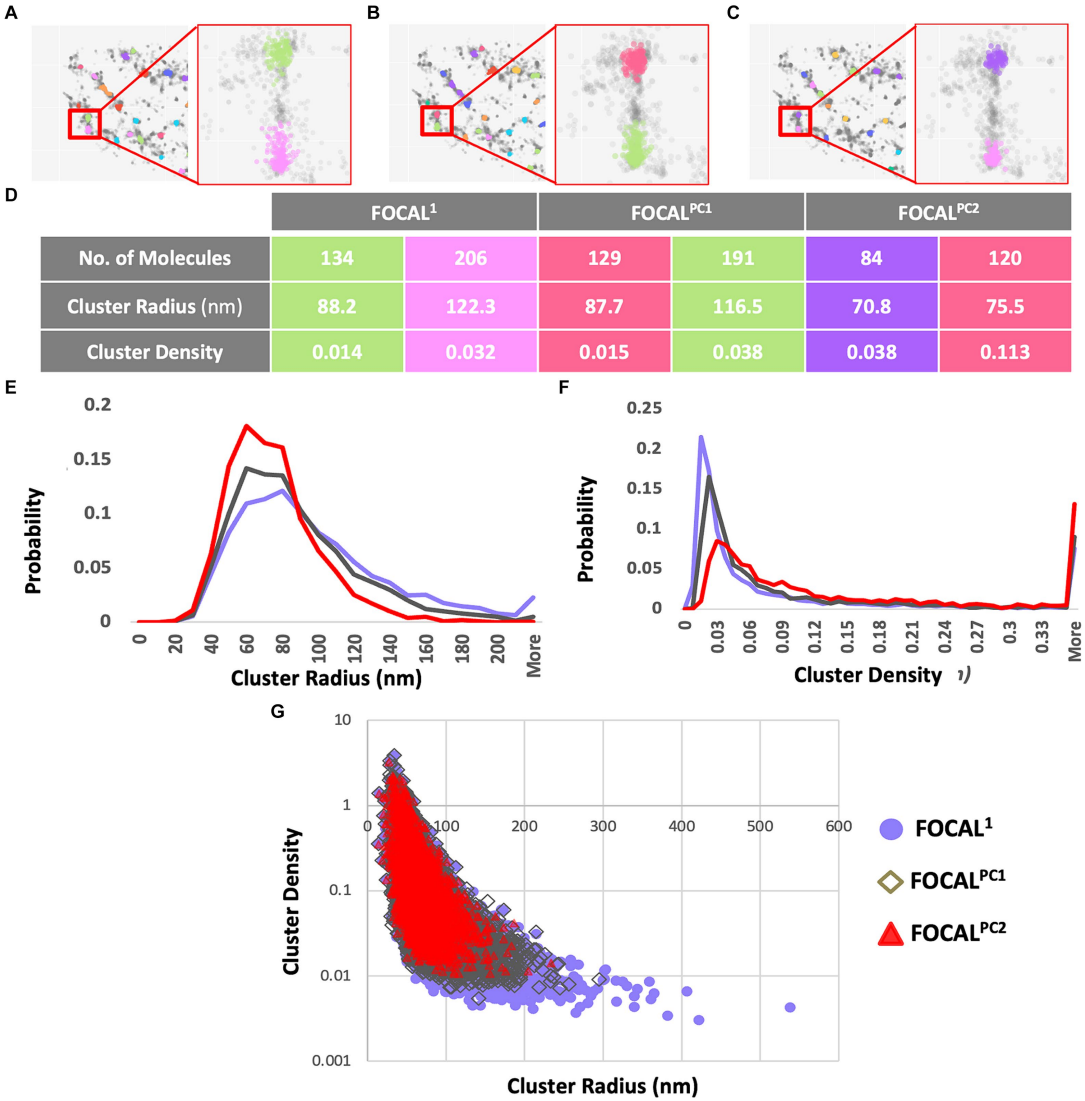
Aggregates’ radii decrease and aggregates’ densities increase when increasing *maPC* from 0 to 2,500 to 5,000 using FOCAL^1^, FOCAL^PC1^, and FOCAL^PC2^. **(A–C)** Clustering visualization and parameters comparison. Aggregate identification in the same area is shown using FOCAL^1^ **(A)**, FOCAL^PC1^ **(B)**, and FOCAL^PC2^ **(C)**. Inserts show the same 2 aggregates under the different FOCAL analyses. **(D)** Aggregate properties, such as the number of localizations in the aggregates, its radius, and density are listed for the 2 aggregates in the inserts for the 3 FOCAL parameters shown above. **(E)** Distribution of aggregates’ radii identified by each algorithm. FOCAL^1^ (purple), FOCAL^PC1^ (gray), and FOCAL^PC2^ (red). **(F)** Distribution of aggregates’ densities identified by each algorithm. FOCAL^1^ (purple), FOCAL^PC1^ (gray), and FOCAL^PC2^ (red). **(G)** Scatter plot showing the correlation between aggregates’ radius and density as detected in the different FOCAL analyses. The mean numbers of aggregates per image in FOCAL^1^ [85.79], in FOCAL^PC1^ [78.403], and in FOCAL^PC2^ [55.919]. The mean percentage of localizations identified as aggregates in FOCAL^1^ = 49.1%, in FOCAL^PC1^ = 49.5%, and in FOCAL^PC2^ = 49.6%.

aSyn is known to form aggregates, while p-aSyn is known to be highly enriched in both brain LBs (Spillantini et al., 1997) and in skin aSyn deposits. To characterize t-aSyn and p-aSyn distribution into aggregates in the skin, we next used several analysis algorithms to analyze the distribution of the *d*STORM localization data into aggregates. We have previously implemented DBSCAN to analyze the organization of aSyn into aggregates (Wegrzynowicz et al., 2019; Sade et al., 2023; Sela et al., 2023) and extended these earlier efforts here by introducing FOCAL and FOCAL^PC^ as additional analysis algorithms (Figure 3). Briefly, FOCAL examines the number of localizations per 3D voxel and defines minimal numbers of localizations per voxel and per neighboring voxels. By implementing FOCAL^PC^ that gives weight to the intensity of each localization and hence its localization precision (see Materials and Methods and Supplementary Figure S2 for more details), we were able to integrate only those voxels that measured single aSyn molecules with higher localization precision into an aggregate (Figure 3 and Supplementary Figure S3).

We first examined the effect of adding the *maPC* to p-aSyn aggregate detection in the PD patients dataset. When *maPC* was increased (from 0 in FOCAL^1^ to 2,500 in FOCAL^PC1^, and to 5,000 in FOCAL^PC2^), less voxels were characterized as part of the aggregates and hence, less localizations were allocated to the aggregate. As such, the aggregates were smaller and became denser (compare FOCAL^1^ to FOCAL^PC1^ and FOCAL^PC2^ values; Figure 3). On average, the number of localizations assigned to clusters by FOCAL^1^ was larger (267.29 ± 4.63), as compared to the assignments made by FOCAL^PC1^ (203.69 ± 2.44) or FOCAL^PC2^ (203.68 ± 2.63). Similarly, FOCAL^1^ reported clusters with larger radii (92.5 nm ±0.6), in comparison with those reported by FOCAL^PC1^ (84.12 nm ±0.5) or FOCAL^PC2^ (74.98 nm ±0.46; Figure 3E). Expectedly, we also found clusters of lower densities with FOCAL^1^ (0.02 ± 0.0006) than with FOCAL^PC1^ (0.09 ± 0.002) or FOCAL^PC2^ (0.13 ± 0.003; Figure 3F). On average, 85.8 clusters were identified by FOCAL^1^ [75 (median), 7.9 (SEM)], a slightly smaller number (78 clusters) was classified by FOCAL^PC1^ (69, 5.5), and the smallest number (56 clusters) was classified by FOCAL^PC2^ (47, 3.2). Furthermore, the percentage of localizations assigned to clusters was reduced as the *maPC* increased (FOCAL^1^: 33.9%, FOCAL^PC1^: 30.3%, and FOCAL^PC2^: 21.6%). It is hence apparent that the additional *maPC* parameter has a visible impact on the clusters identified by FOCAL^PC^. It should be noted that the new algorithm gave similar results to the DBSCAN algorithm that was used in a previous study (Wegrzynowicz et al., 2019; Supplementary Figure S4), but decreased the range of cluster densities and radii, as well as the number of identified clusters, thereby making clustering more specific to more dense clusters, presumably representing pathological p-aSyn deposits.

**Figure 4:**
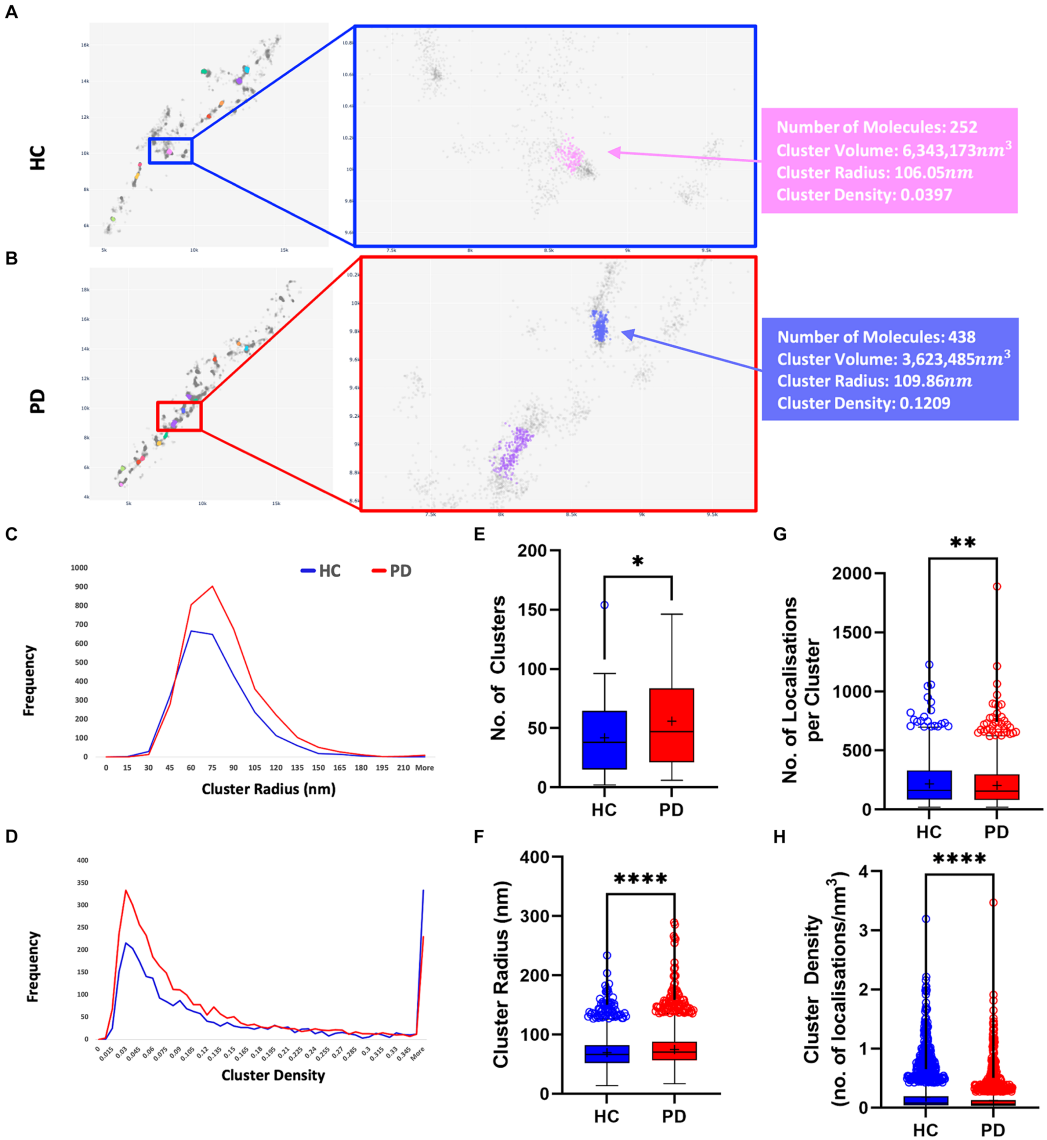
PD patients display a higher number of aggregates compared to HC subjects. **(A,B)** Visualization of aggregates identified by FOCAL^PC2^ in an HC subject **(A)** and a PD patient **(B)**. **(C)** Distribution of aggregates’ radii shows that PD patient-derived images contain a larger number of aggregates with medium radii than do HC subject-derived images. **(D)** Distribution of aggregates’ densities shows that PD patient-derived images display a larger number of aggregates with low-medium densities. **(E)** Number of aggregates is significantly higher in PD patients than in HC subjects [means – HC: 42.45, PD: 55.92]. **(F)** Radii of aggregates are significantly larger in PD patients than in HC subjects [means – HC: 69.43, PD: 74.98]. **(G)** Densities of aggregates are significantly higher in HC subjects than in PD patients [means – HC: 0.1785, PD: 0.1263]. **(H)** Number of localizations per aggregates is significantly higher in HC subjects than in PD patients [means – HC: 217.66, PD: 203.68]. The mean percentage of localizations identified as aggregates in HC subjects is 20.3% and in PD patients is 21.6%. Means are marked with a + sign, and the 25^th^ percentile, median, and 75th percentile are marked by the bottom, middle, and top black lines outlining the whisker box, respectively. Each outlier data point is marked with a diamond. *p < 0.05, **p < 0.01, ****p < 0.0001.

**Figure 5:**
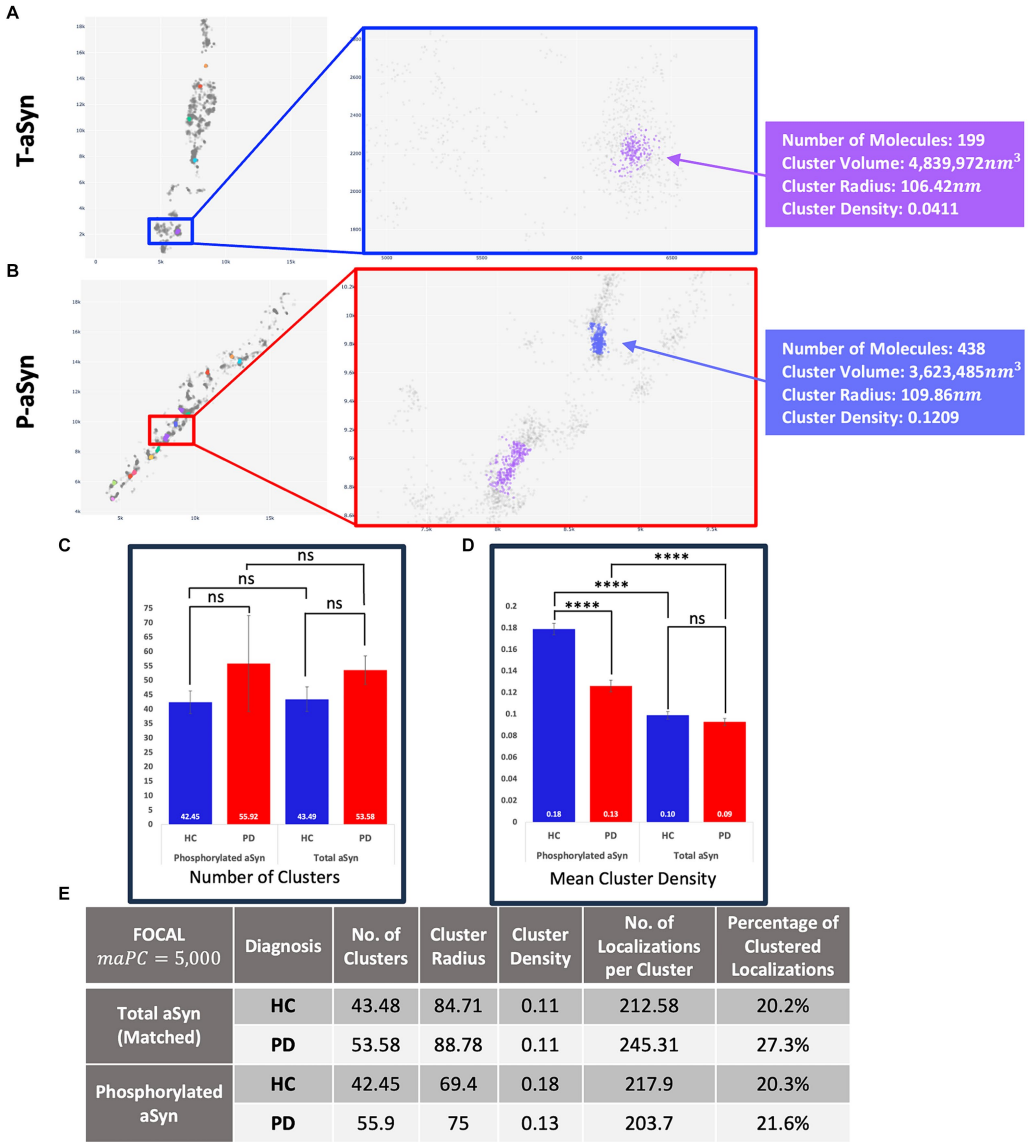
t-aSyn aggregates are larger and less dense compared to p-aSyn aggregates in both PD patients and HC subjects. Comparison of t-aSyn and p-aSyn clustering in PD patients and HC subjects using FOCAL^PC2^. **(A,B)** Visualizations of clusters of t-aSyn **(A)** and p-aSyn in a PD patient **(B)**. **(C–E)** t-aSyn aggregates are larger and less dense compared to p-aSyn aggregates for both PD patients and HC subjects. ****p < 0.0001.

Figures 4, 5 present the distribution of p-aSyn aggregates and t-aSyn in PD patients and HC subjects as obtained with the modified FOCAL. We hypothesized that the number or composition of p-aSyn aggregates would differ between PD patients and HC subjects. The average number of p-aSyn clusters per image identified by FOCAL^PC2^ was significantly larger in PD patients (56 ± 4.705) than in HC subjects (42 ± 3.914; Figure 4E). The total number of p-aSyn aggregates found and analyzed in PD patients (3,467) was larger than the total number of p-aSyn aggregates found in HC subjects (2,547). We found no effect of the subjects’ age on the number of clusters either in HC or in PD (Supplementary Figure S6). p-aSyn clusters were significantly larger in PD patients (75 nm ±0.459) than in HC subjects (69 nm ±0.487; Figures 4C,F) but were significantly denser in HC subjects (0.18 localizations per nm^3^ ± 0.005) than in PD patients (0.13 localizations per nm^3^ ± 0.004; Figures 4D,H). In agreement with these observations, the clusters identified by FOCAL^PC2^ in PD patients (203.68 ± 2.626) contained fewer localizations than those in HC subjects (217.66 ± 3.23, Figure 4G). Hence, in agreement with our hypothesis, skin slices of PD patients contained more p-aSyn aggregates than those of HC subjects.

We then compared and characterized the t-aSyn-containing clusters detected. Similar to the analysis of p-aSyn, PD patients contained more t-aSyn aggregates per image (53.58 ± 4.97) than HC subjects (43.49 ± 4.27; Figure 5). Cluster radii were significantly smaller in HC subjects (84.71 ± 2.19) than in PD patients (88.78 ± 2.42) and their densities were not significantly different between HC subjects (0.11 $\frac{\mathrm{localizations}}{nm^{3}}$ ±0.0099) and PD patients (0.11 $\frac{\mathrm{localizations}}{nm^{3}}$ ±0.008). However, t-aSyn clusters were slightly sparser than p-aSyn (Figures 5C,E). This might reflect differences between pathological p-aSyn versus physiological t-aSyn. Most probably, the t-aSyn antibody also recognizes the p-aSyn in the synapse, which is organized in aggregates. The findings thus provide additional support that *d*STORM combined with immuno-labeling using different anti-aSyn antibodies allows the detection of both the physiological and pathological form of aSyn.

## Discussion

In this study, we introduced an innovative integration of SRM with an advanced analysis platform, enhancing both detection sensitivity and the information extracted from images. This integrated approach facilitated comprehensive profiling of nanoscale aSyn aggregates in skin biopsies obtained from both PD patients and HC subjects. Our novel strategy allowed for aggregate characterization based on various quantitative criteria, such as aggregate size, distribution, and density—information that was not previously available using convectional microscopy, or SAA. Our findings suggest that p-aSyn, representing the pathological form of aSyn, is organized into dense aggregates, sized approximately 75 nm, and that these aggregates are more enriched in PD patients than in HC subjects.

Today, by the time PD is clinically diagnosed, an irreversible loss of approximately 70–80% of dopaminergic neurons at the substantia nigra has already occurred (Sveinbjornsdottir, 2016), leaving little room for intervention of the basic pathological process responsible for the neurodegenerative process. PD diagnosis based on quantitative parameters thus represents an unmet need that offers a route to revolutionize the way PD and other synucleinopathies are diagnosed and treated (Hughes et al., 2001, 2002; Tolosa et al., 2006). aSyn-SAA can differentiate between different HC and PD patients and between different synucleinopathies, such as PD and multiple systems atrophy (MSA) (Manne et al., 2020; Shahnawaz et al., 2020; Kuzkina et al., 2021; Mammana et al., 2021; Concha-Marambio et al., 2023; Iranzo et al., 2023; Donadio et al., 2024; Gibbons et al., 2024; Huang et al., 2024). In a new approach used to define PD and dementia with LBs based on a biological definition, it was suggested that aSyn-SAA of CSF can provide a reliable measure of the status of pathological processes associated with aSyn (Simuni et al., 2024). Still, SAA does not provide information on different aSyn species, their distribution, density, and size, and at the moment, it does not provide a quantitative measure that correlates with disease progression. Hence, the application of SRM and advanced analysis to PD diagnosis provides these missing quantitative parameters, namely, aggregate size, distribution, density, and shape. This can also be used in the future to track disease progression quantitatively. Furthermore, SRM analysis of a skin biopsy can provide information about the patient’s molecular status in a much less invasive manner, as compared to a lumbar puncture for collecting CSF.

Previous studies suggested that detecting aSyn aggregates in skin biopsies holds good promise for PD diagnosis (Al-Qassabi et al., 2020; Mammana et al., 2021; Iranzo et al., 2023; Waqar et al., 2023; Donadio et al., 2024; Gibbons et al., 2024), yet thus far it has not met the requirements needed to become clinically relevant. In most cases, the immunoassays that are employed overlook the size, density, and composition of aSyn aggregates. We hypothesized that these parameters are essential for understanding synuclein pathology in PD and should be examined as well. The application of SRM methods for addressing various diseases (Suleiman et al., 2013), some associated with protein aggregation, provides new mechanistic information that allows both insight into disease state and progression (Zhang et al., 2015; Wegrzynowicz et al., 2019; Padmanabhan et al., 2021; Johansson et al., 2023) and into the mechanism of drug action (Wegrzynowicz et al., 2019; Sela et al., 2023). For example, using SRM to study the aggregate species secreted from cells, it was found that seeding of externally applied fibrils resulted in the increased secretion of nanoscopic aSyn and Aβ aggregates with a mean diameter of 35 nm (Sang et al., 2003). As conventional confocal microscopy cannot differentiate between the aggregates sized below 200 nm (i.e., any aggregate between 10 and 200 nm will be seen as a 200 nm aggregate), SRM allowed the identification of these sub-diffraction aggregates for the first time. Hence, the use of SRM allows the differentiation of these small aggregates and the ability to define what aggregate species are secreted. Similarly, it was shown that small (20–300 nm), rather than large (>500 nm) aSyn aggregate levels correlated with disease progression in a mouse model of PD (Wegrzynowicz et al., 2019), again emphasizing the strength of SRM for examining sub-diffraction aggregates. The current study provides new information on the arrangement of aSyn aggregates in the innervation of PD patients’ skin.

Using a tailor-made in-house platform termed ‘dSTORM Analyser’ that is available to the scientific community, we created a novel platform that revealed unique fingerprints of aSyn aggregates. The analysis detected a larger number of clusters, clusters with larger radii, sparser clusters, and clusters containing a smaller number of localizations in PD patients, relative to what was seen with HC subjects. These findings provide complementary information to SAA results by providing new characteristics of the aggregates. No correlation was detected between the number of clusters and the participants’ ages. However, a larger patient cohort, with a smaller variability in participants’ ages, and longitudinal tracking of the changes in clusters properties along the progression of the disease will be beneficial in future studies. (Kuzkina et al., 2021, 2023; Wang et al., 2021). The study also shows that p-aSyn in PD patients is organized into aggregates with an average radius of 75 nm and that the ratio of neuronal staining to aSyn expression is higher in HC than in PD. This suggests that the existence of more p-aSyn correlates with reduced nerve fibers in PD patients. Hence, our study offers new quantitative biomarkers for future comparison of various aSyn aggregates (Young et al., 2019; Doppler, 2021).

Most studies that utilize immunoassays for p-aSyn detection reported that HC subjects did not contain p-aSyn. Yet, our results clearly showed p-aSyn molecules in skin biopsies from HC subjects. Although our efforts relied on regular antibody staining, we were able to detect every single p-aSyn molecule, thus providing evidence that HC also contains p-aSyn molecules, unlike what was reported in other studies. Still, it was previously suggested that diffuse or granular p-aSyn staining is found in HC (Al-Qassabi et al., 2020; Waqar et al., 2023). This could explain the staining that we detected in HC subjects, given how *d*STORM is more sensitive and detects every molecule, demonstrating that p-aSyn molecules are found in HC subjects. It is also possible that the p-aSyn antibody is less specific than advertised, and it also recognizes some of the t-aSyn molecules. Yet, the detailed *d*STORM image analysis revealed that in PD patients, p-aSyn molecules are organized into aggregate-like structures, with this being less frequent in HC. This correlates with previous publications that suggested that discrete p-aSyn aggregates were found in PD patient biopsies (Al-Qassabi et al., 2020; Waqar et al., 2023).

An unmet need in treating PD is the availability of a sensitive, quantitative, and specific biomarker that could serve to report on the progression of pathological processes in the early stages of the disease. Such a sensitive and reliable biomarker will allow subjects at risk to receive disease-modifying treatment at the prodromal stages and potentially prevent the clinical syndrome of PD altogether. At present, many novel technologies are being tested in advanced clinical stages, aiming to intervene in the basic pathological process responsible for PD. Such technologies include those leading to the inhibition of aSyn aggregation, reduction of aSyn expression, reduction in *leucine-rich repeat kinase 2* (LRRK2) kinase activity, enhancement of glucocerebrosidase activity, and stem cell transplantation (Chopade et al., 2023; McFarthing et al., 2023). Ideally, subjects at risk, such as carriers of specific mutations in the glucosylceramidase beta 1 (GBA1) or LRRK2 genes or subjects with prodromal symptoms such as rapid eye movement (REM) sleep without atonia or decreased sense of smell, could undergo a simple procedure to extract a skin biopsy, undergo full SRM aggregate analysis, and potentially be treated (if positively diagnosed with the pathological biomarker for PD) before irreversible neural damage has occurred.

To conclude, using rigorous detection of *d*STORM together with our advanced ‘dSTORM Analyser’ platform, we were able to characterize aSyn aggregate traits in skin biopsies for the first time. In future research, correlating the molecular fingerprints identified here with patient clinical status and disease progression may provide a new molecular toolbox that can be used to (a) track disease progression at the pathological level objectively, (b) track the efficacy of disease-modifying treatment, and (c) set a new threshold for PD patients with defined aSyn aggregates levels for recruitment to clinical trials, thus reducing patient population heterogeneity. Furthermore, the implementation of our unique method will advance our understanding of the aSyn aggregation process and may define peripheral aSyn as a reliable biomarker for following PD pathology and disease progression.

## Data availability statement

All datasets will be available upon request to the email ofirsade@mail.tau.ac.il.

## Ethics statement

The studies involving humans were approved by Ethics Committees of Tel-Aviv Sourasky Medical Center, Meir Medical Center and Sheba Medical Center (8492-21, 0257-08 and 0302-23). The studies were conducted in accordance with the local legislation and institutional requirements. The participants provided their written informed consent to participate in this study.

## Author contributions

OS: Conceptualization, Formal analysis, Investigation, Methodology, Software, Writing – original draft, Writing – review & editing. DF: Formal analysis, Investigation, Methodology, Writing – review & editing. NB-B: Investigation, Methodology, Writing – review & editing. SH: Investigation, Methodology, Writing – review & editing. IG: Supervision, Writing – review & editing, Writing – original draft. DB-O: Writing – review & editing, Conceptualization. SFS: Methodology, Writing – review & editing. AM: Writing – review & editing, Resources. AT: Writing – review & editing, Resources. AG: Resources, Writing – review & editing. MK: Resources, Writing – review & editing. MG: Writing – review & editing, Resources. SA: Resources, Writing – review & editing. CS: Writing – review & editing. MSR: Writing – review & editing. SS: Statistics expertise, Writing – review & editing. KD: Methodology, Writing – review & editing. MS: Writing – review & editing, Methodology. NG: Writing – review & editing, Resources, Supervision. NL: Writing – review & editing, Resources, Supervision. RA: Writing – review & editing, Resources, Supervision. SH-B: Writing – review & editing, Resources, Supervision. UA: Writing – original draft, Writing – review & editing, Conceptualization, Methodology.
