# Supplementary material for: A novel super-resolution microscopy platform for cutaneous alpha-synuclein detection in Parkinson’s disease
Source: Front Mol Neurosci. 2024 Sep 4;17:1431549. doi: 10.3389/fnmol.2024.1431549 (PMC11409901; doi:10.3389/fnmol.2024.1431549)

Supplementary Material


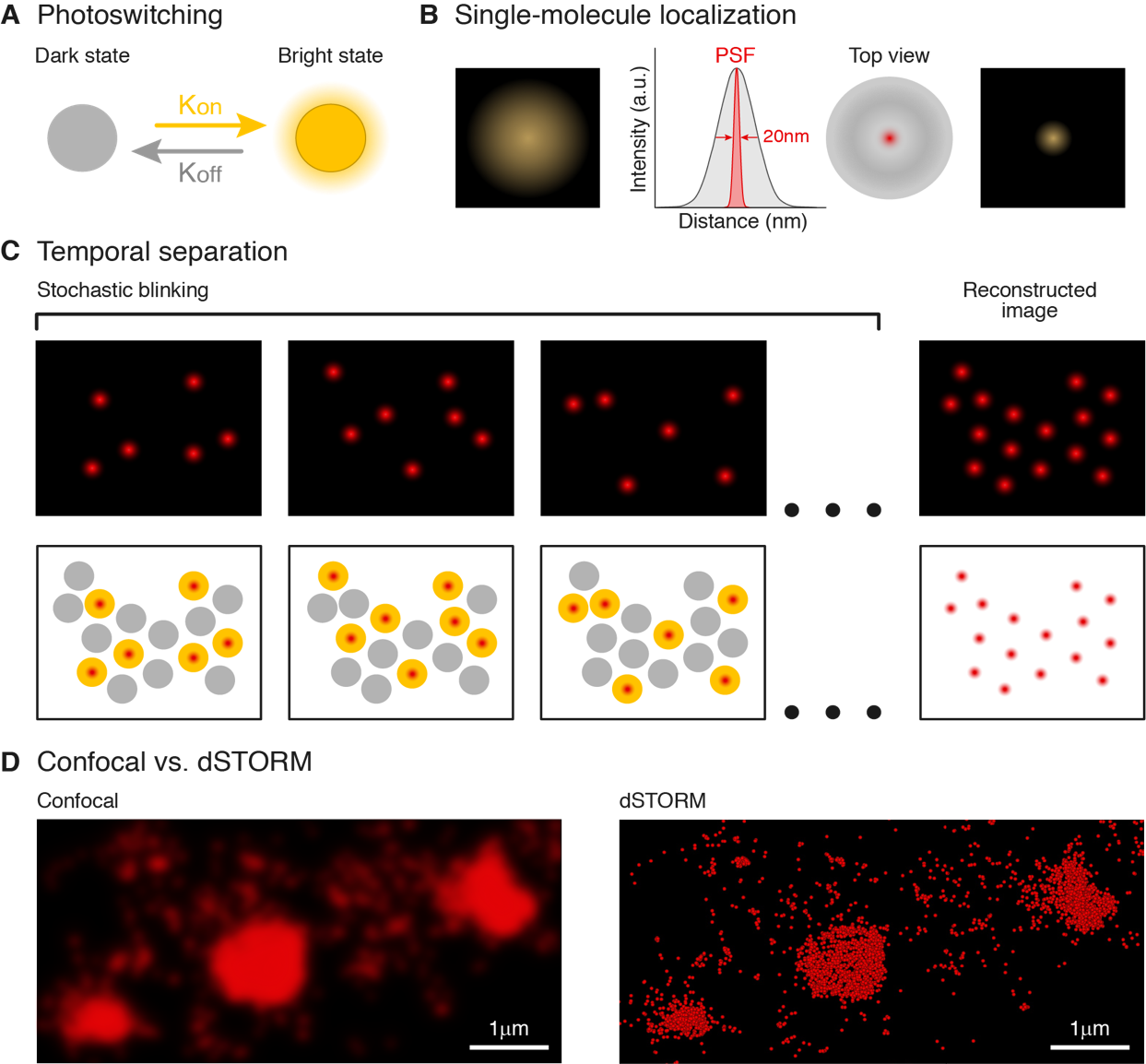


**(A)**

**(B)**

**(C)**

**(D)**

**Supplementary Figure 1. Depiction of the *d*STORM imaging method. A.** Fluorophores are photo-switched between bright and dark states. **B.** The position of a single fluorophore (i.e., a molecule) is estimated by its PSF achieving to precisely localized each molecule (red dot) with 10-20nm spatial resolution. **C.** Each sample is imaged for 3,000 frames and in each frame, stochastic activation of different molecules occurs (upper panel), PSFs are fitted to each blink to define its exact localization (lower panel) and a reconstruction image of all the localizations is formed (right image)**. D.** *d*STORM (right) allows improved resolution (~10-20 nm), as compared to confocal microscopy (~200 nm, left) and enables detection of single molecule and of subdiffractional aggregates sub-populations, which cannot be otherwise distinguished.

| **Participant Number** | **Age** | **Sex** | **Diagnosis** | **T.F.D.** | **H&Y** | **UPDRS-motor** | **Scopa-Autonomic** | **MoCA** |
| --- | --- | --- | --- | --- | --- | --- | --- | --- |
| **1** | 68 | M | PD | 1 | 2 | 27 | 15 | 25 |
| **2** | 76 | M | PD | 12 | 2 | 39 | 13 | 19 |
| **3** | 68 | M | PD | 37 | 2 | 27 | 4 | 24 |
| **4** | 71 | F | PD | 132 | 2.5 | 21 | - | 27 |
| **5** | 60 | M | PD | 24 | 2 | 22 | 6 | 27 |
| **6** | 77 | F | PD | 23 | 1 | 41 | 4 | 23 |
| **7** | 80 | M | PD | 42 | 4 | 62 | 14 | 21 |

**(B)**

**(A)**

| **Participant Number** | **Age** | **Sex** | **Diagnosis** | **T.F.D.** | **H&Y** | **UPDRS-motor** | **Scopa-Autonomic** | **MoCA** |
| --- | --- | --- | --- | --- | --- | --- | --- | --- |
| **1** | 69 | M | HC | 0 | 0 | 0 | 4 | 28 |
| **2** | 56 | F | HC | 0 | 0 | 0 | 0 | 30 |
| **3** | 33 | M | HC | 0 | 0 | 0 | 0 | 25 |
| **4** | 75 | M | HC | 0 | 0 | 0 | 12 | 25 |
| **5** | 72 | M | HC | 0 | 0 | 4 | 0 | 22 |
| **6** | 62 | M | HC | 0 | 0 | 0 | 10 | 29 |
| **7** | 62 | F | HC | 0 | 0 | 2 | 0 | 27 |

**Supplementary Table 1. General information on healthy control biopsy donors (A) and Parkinson’s disease biopsy donors (B) that were immuno-stained for p-aSyn and t-aSyn.** Skin sections from PD subjects 1,4,5,6 were stained for t-aSyn, and 1-7 for p-aSyn. Skin sections from HC subjects 3-6 were stained for t-aSyn, and 1-7 were stained for p-aSyn. It includes, age, gender, diagnosis, Time from diagnosis (T.F.D.) is measured in months, values for UPDRS-motor, Scopa Autonomic and MoCA.


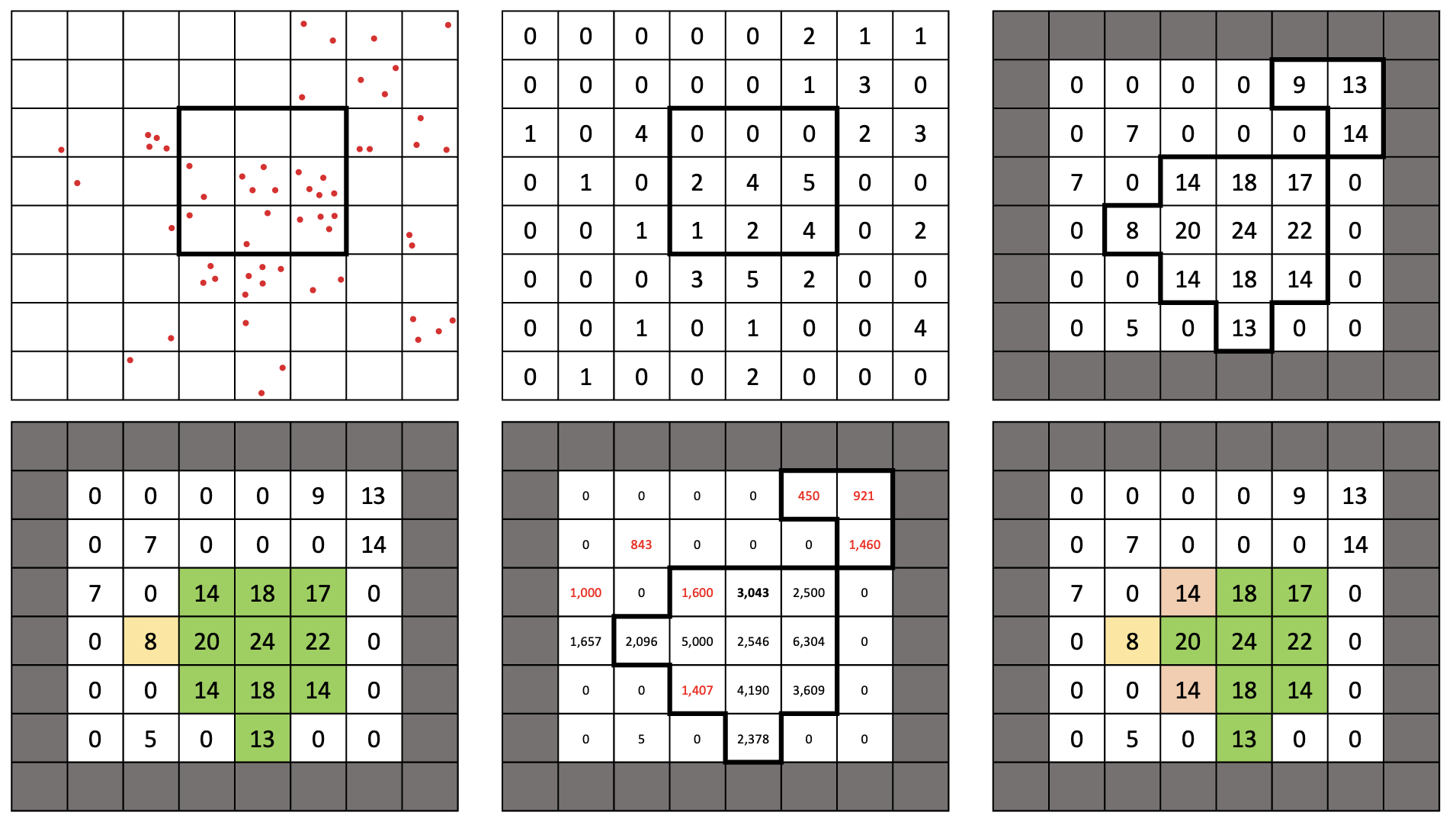

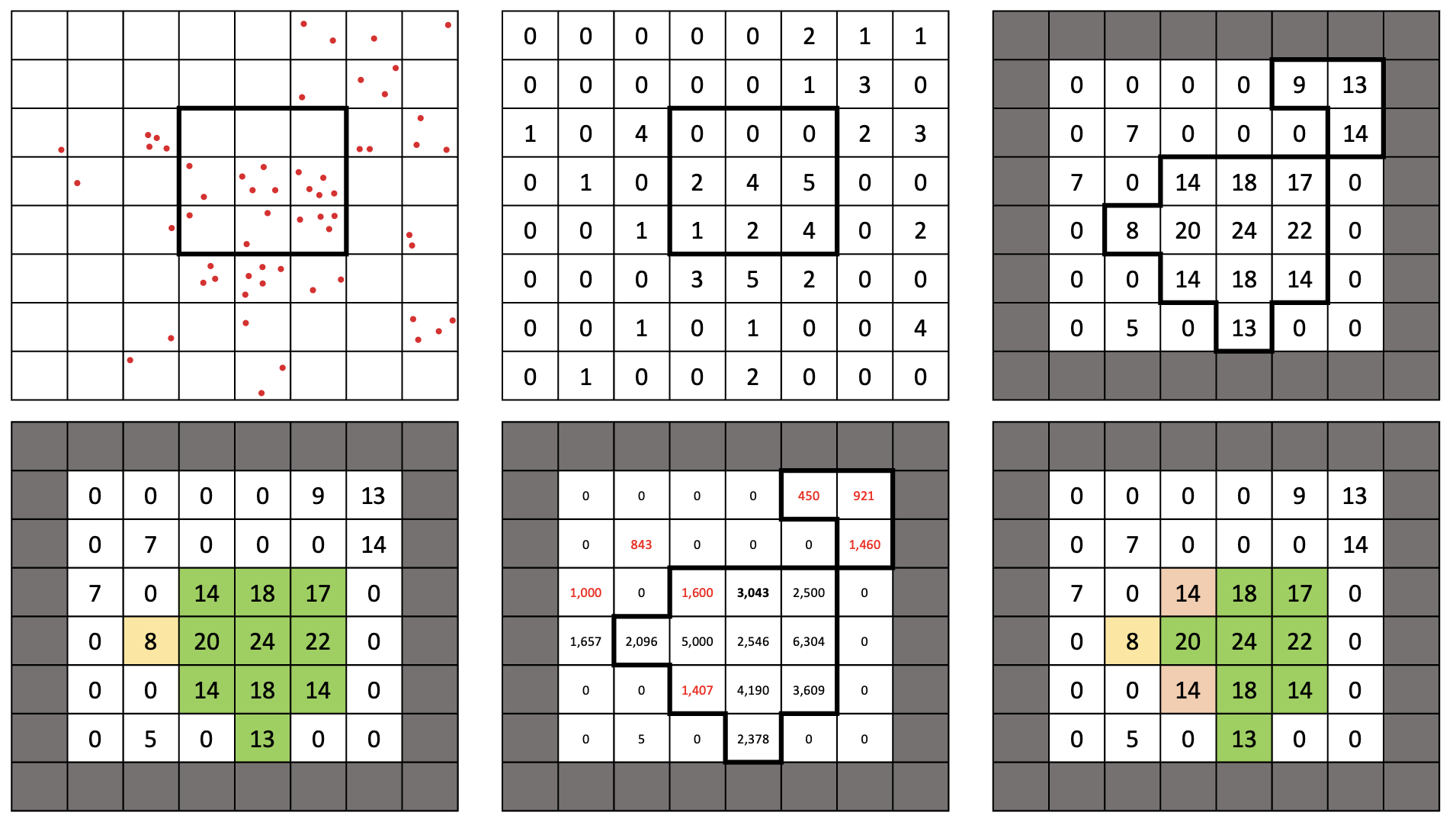

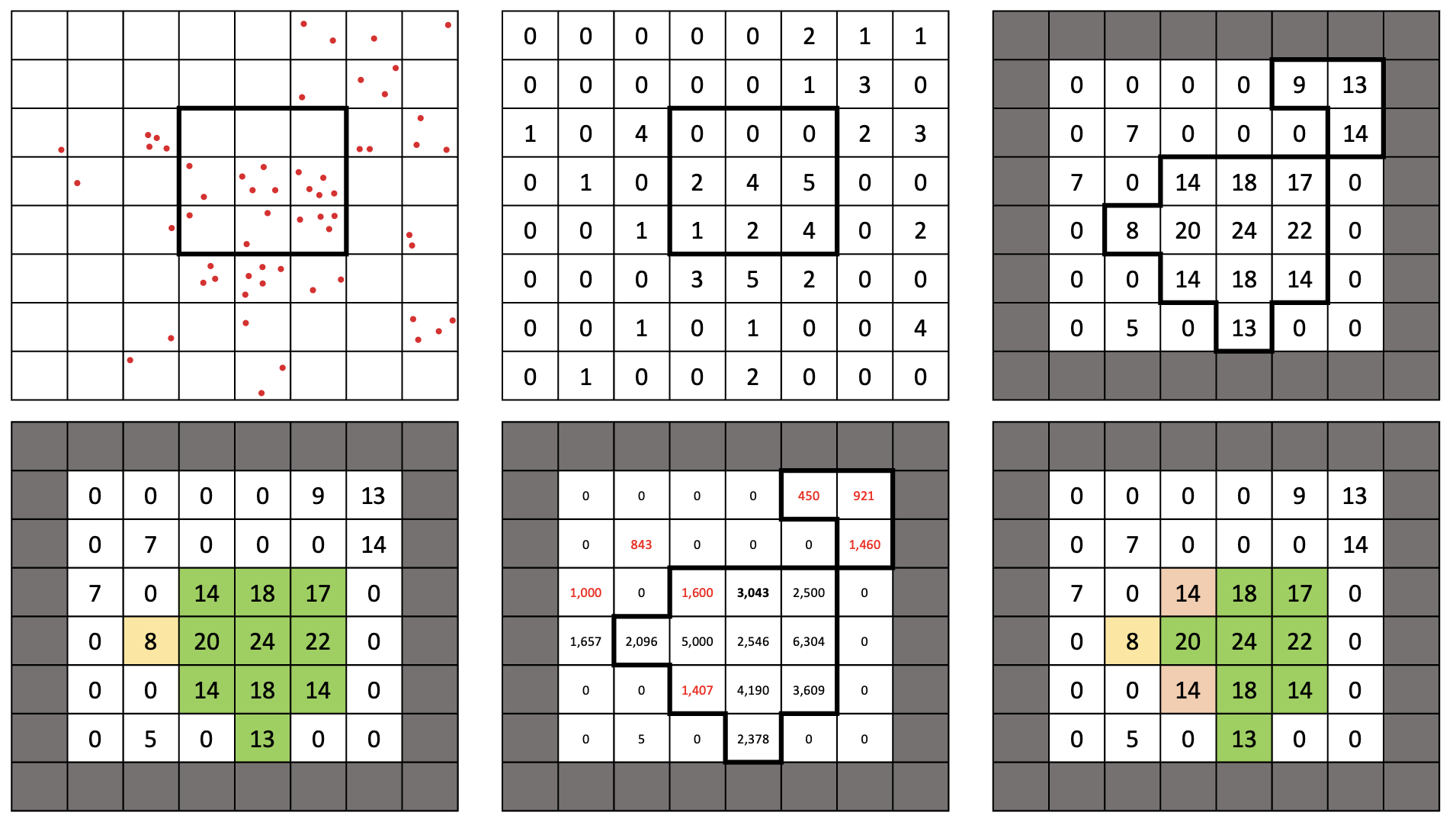


**(A)**

**(B)**

**(C)**

**(D)**

**(E)**

**(F)**

**Supplementary Figure 2. FOCAL clustering algorithm demonstration. A.** An Illustration of points (molecules) distribution**. B.** The number of points in each bin sized $\sigma\times\sigma$ was calculated. $\boldsymbol{\sigma=3}$. **C.** A density map was constructed by assigning each bin a score of the sum of the number of points of a $3\times3$ 3D array surrounding that bin. Bins at the edge of the image (shaded in grey) were discarded from analysis. A density threshold ($minL$) was set to 9 for the purposes of this example, which specifies core (bolded) and border bins. **D.** In the original implementation of FOCAL, only clusters of minimum size $minC$ (in this example $minC=3\times3+1$) would have been retained to filter single localization sources. The identified cluster is shown in green and yellow representing core and border bins, respectively. **E.** Neighbouring core bins were given an additional score of the average photon count of the localizations they contain. Bins that have a lower minimum average photon count ($maPC$) were considered as border bins from this point on. The average photon count threshold ($maPC$) was set to 2,500 for the purposes of this example. **F.** Only clusters of minimum size $minC$ (in this example $minC=3\times3+1$) which passed the $maPC$ threshold were retained to filter single localization sources. The identified cluster is shown in green and yellow border bins while orange bins represent previously core bins that are now excluded due to low $maPC$. FOCAL is presented in 2D for simplicity. Parameters: $\sigma=3$, $minL=9$, $minC=10, maPC=2,500$


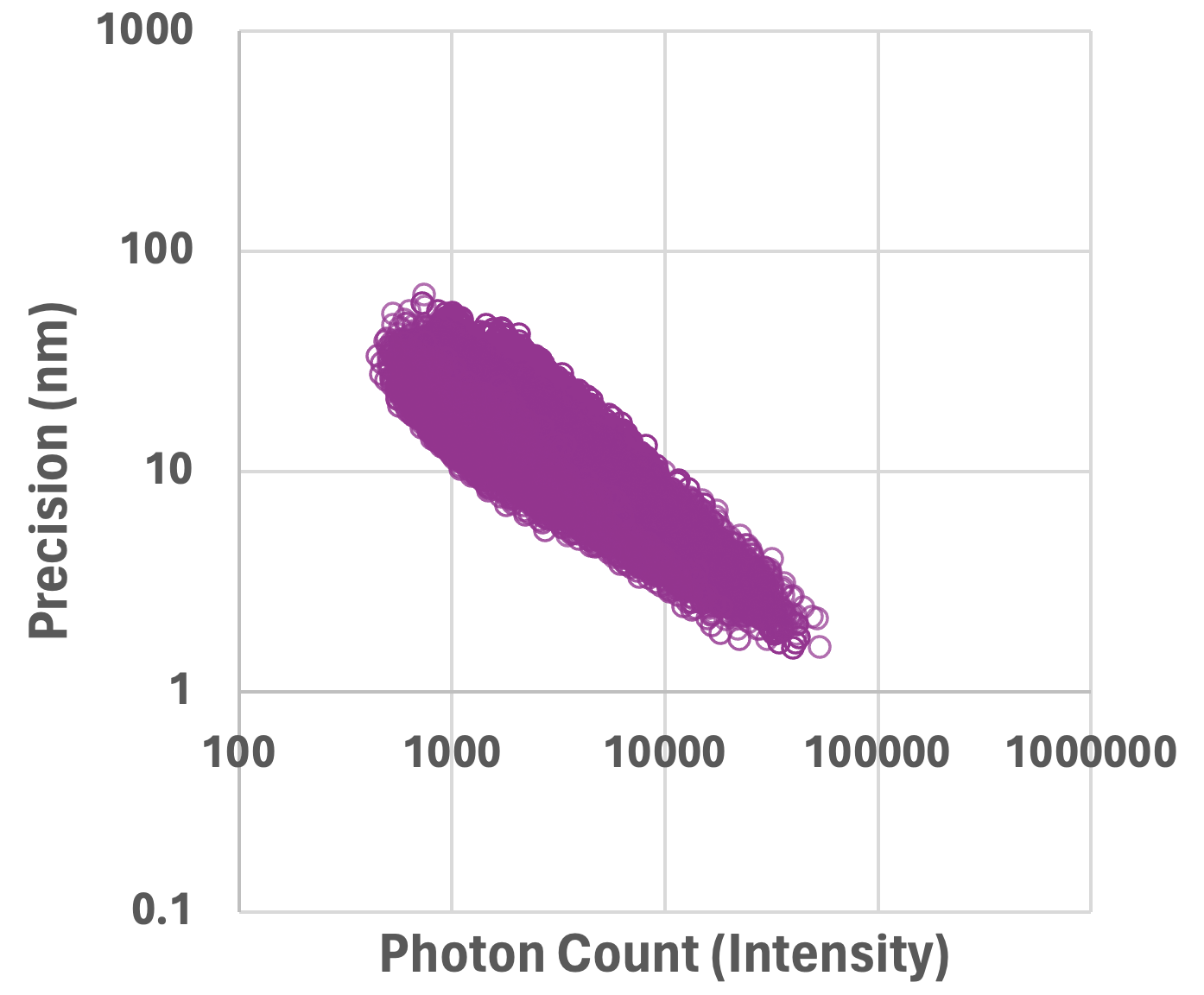

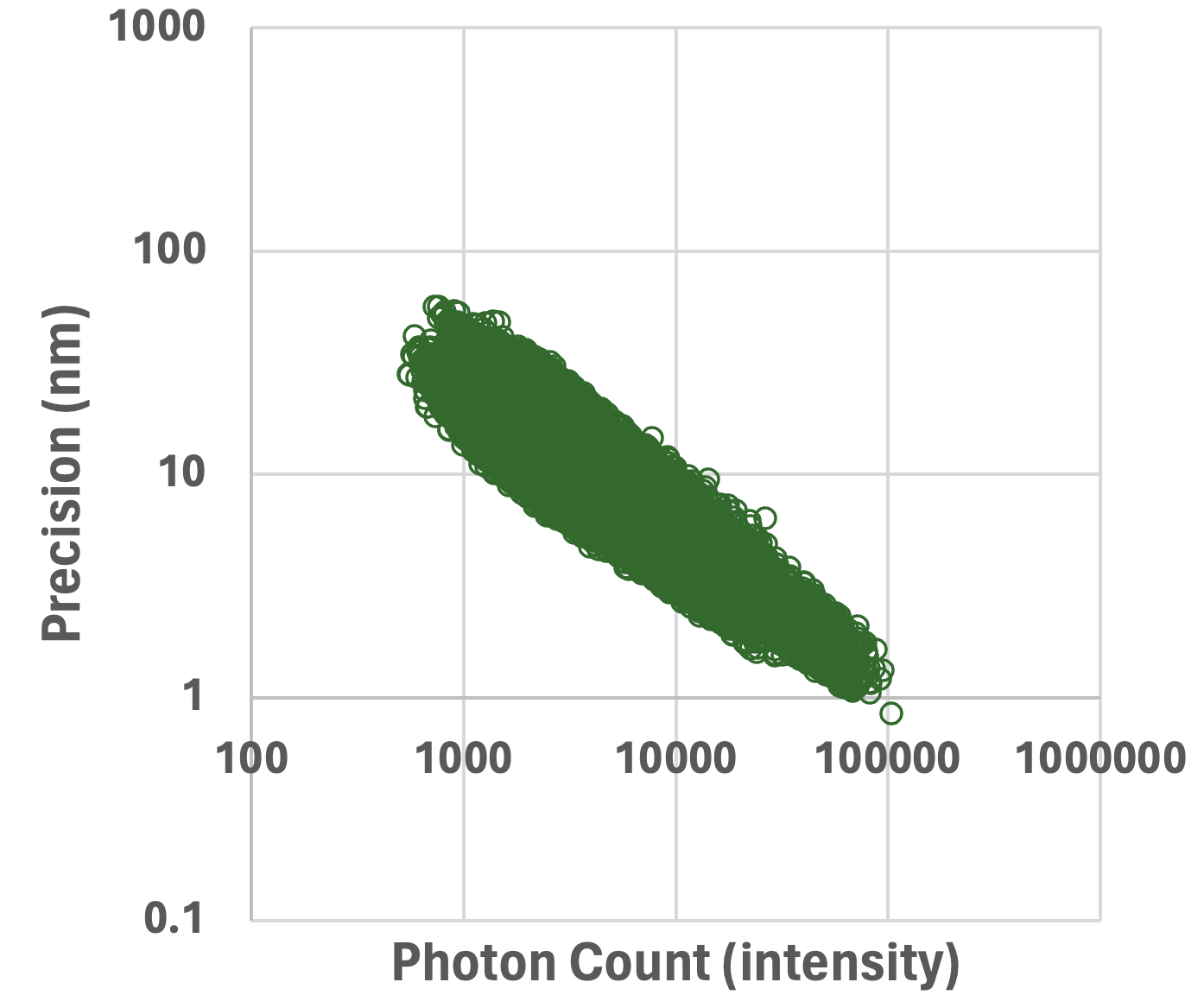

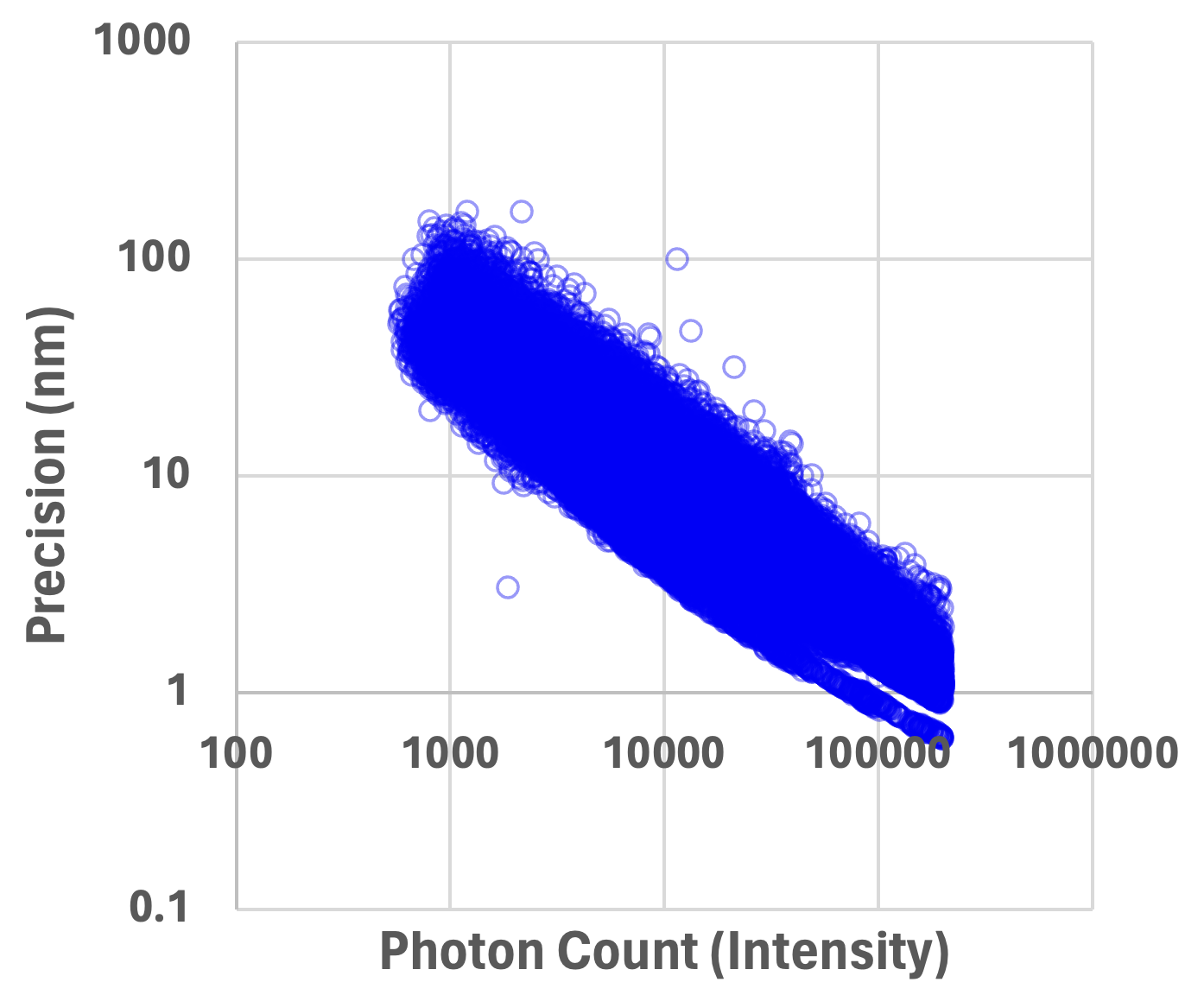

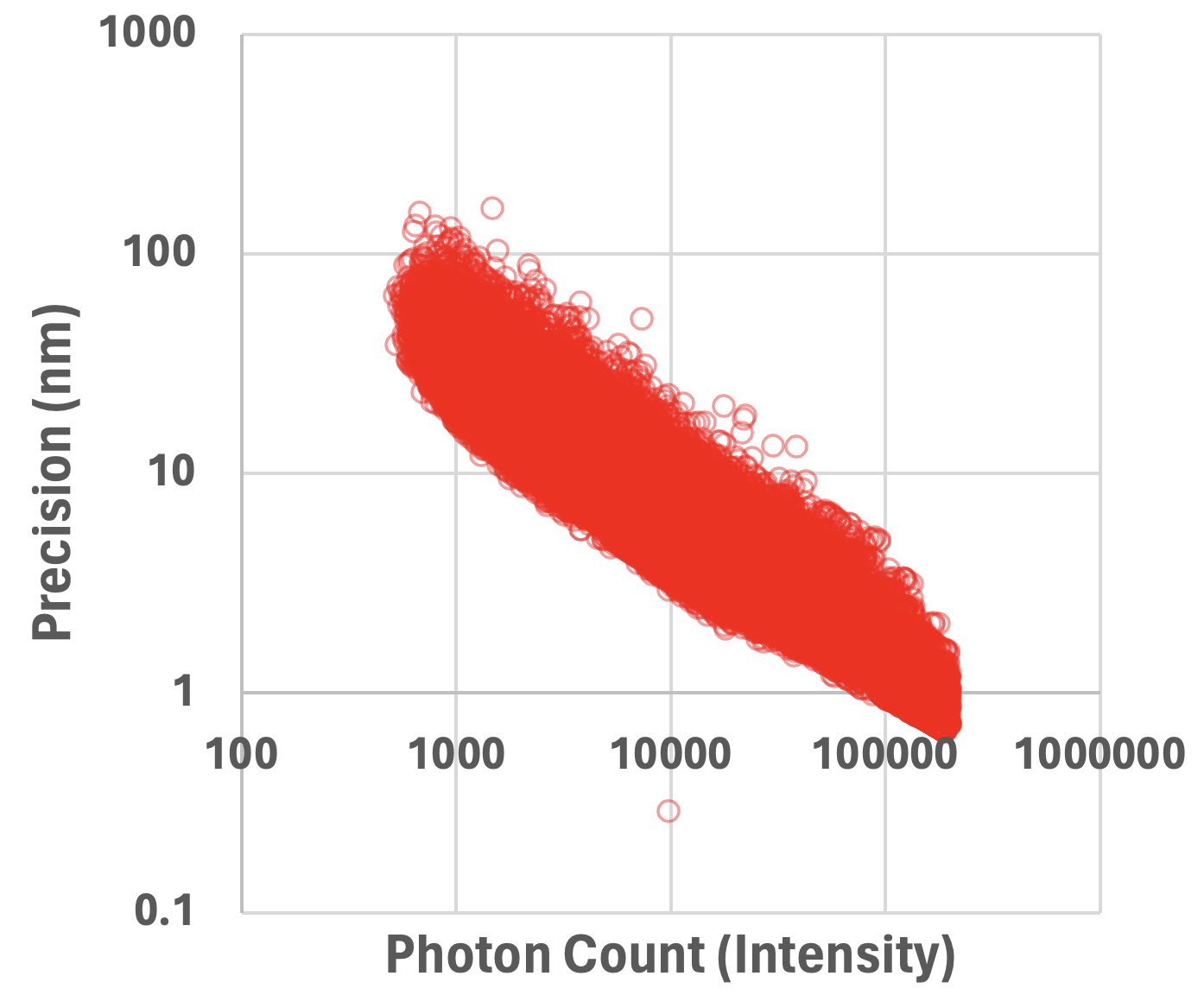


**Total-aSyn**

**Phosphorylated-aSyn**


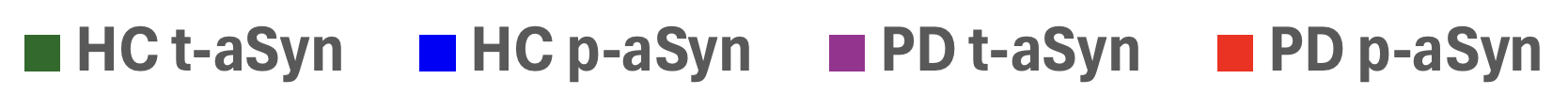

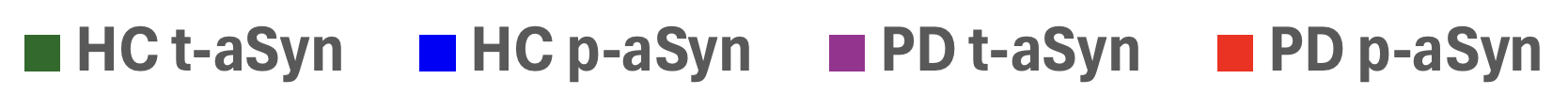

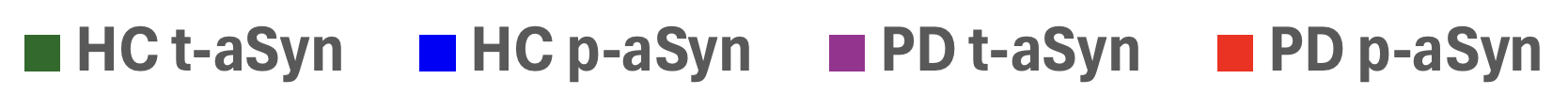

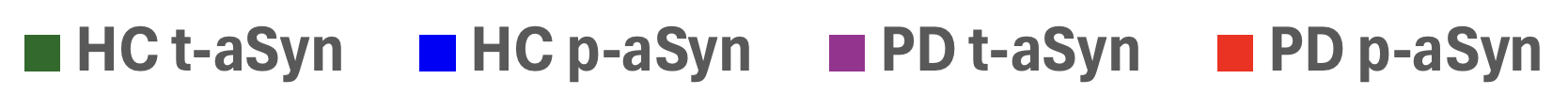


**(B)**

**(A)**

**(C)**

**(D)**

**(E)**


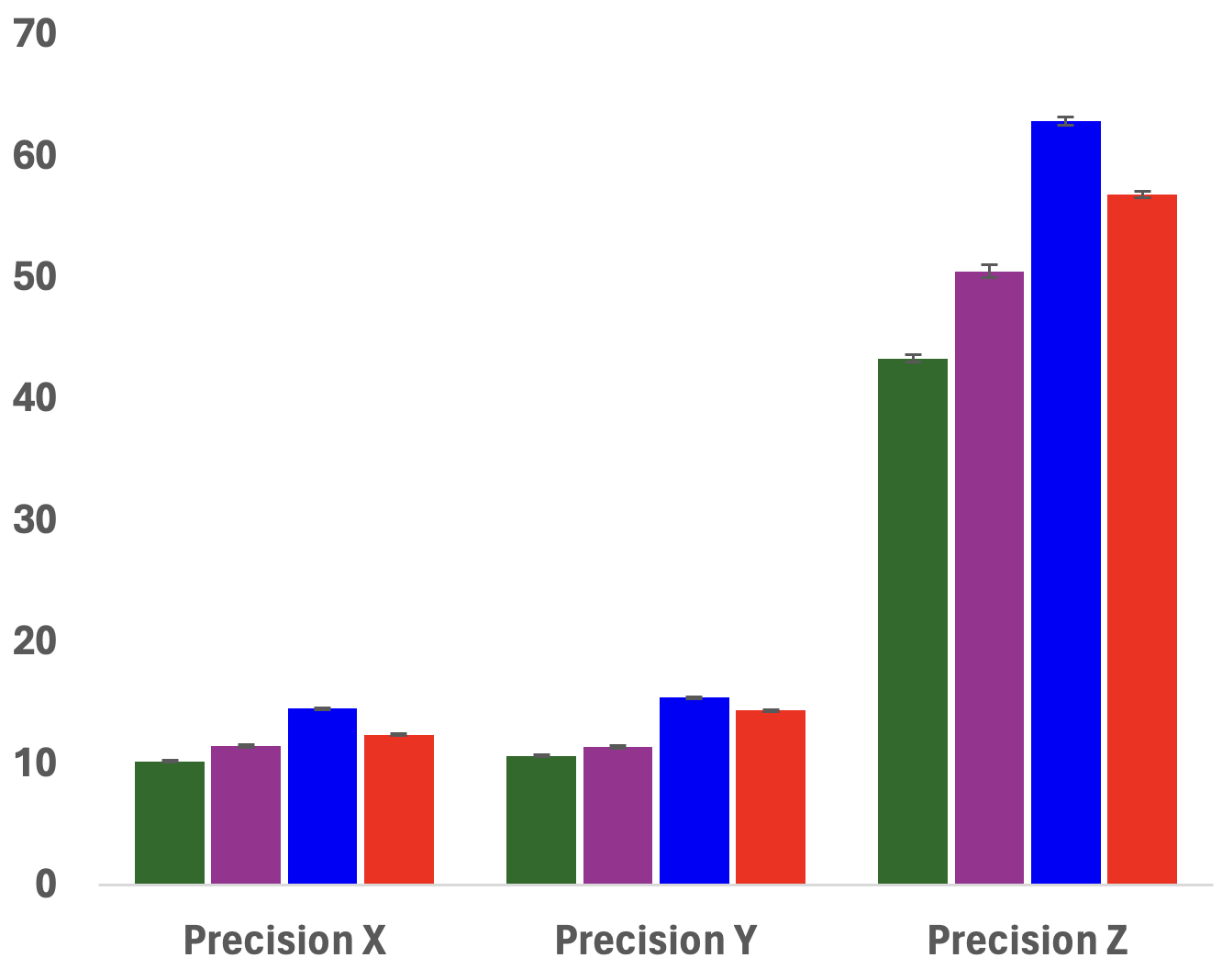


**Supplementary Figure 3. A correlation between photon count and localization precision in randomly sampled *d*STORM images show that localization with high PC has better spatial precision. A.** HC t-aSyn [$r_{S}=-0.948$]. **B.** PD t-aSyn [$r_{S}=-0.935$]. **C.** HC p-aSyn [$r_{S}=-0.962$]. **D.** PD p-aSyn [$r_{S}=-0.954$]. **E.** Mean localization precision in the x, y, and z axes, with standard error of the mean for PD patients and HC subjects.


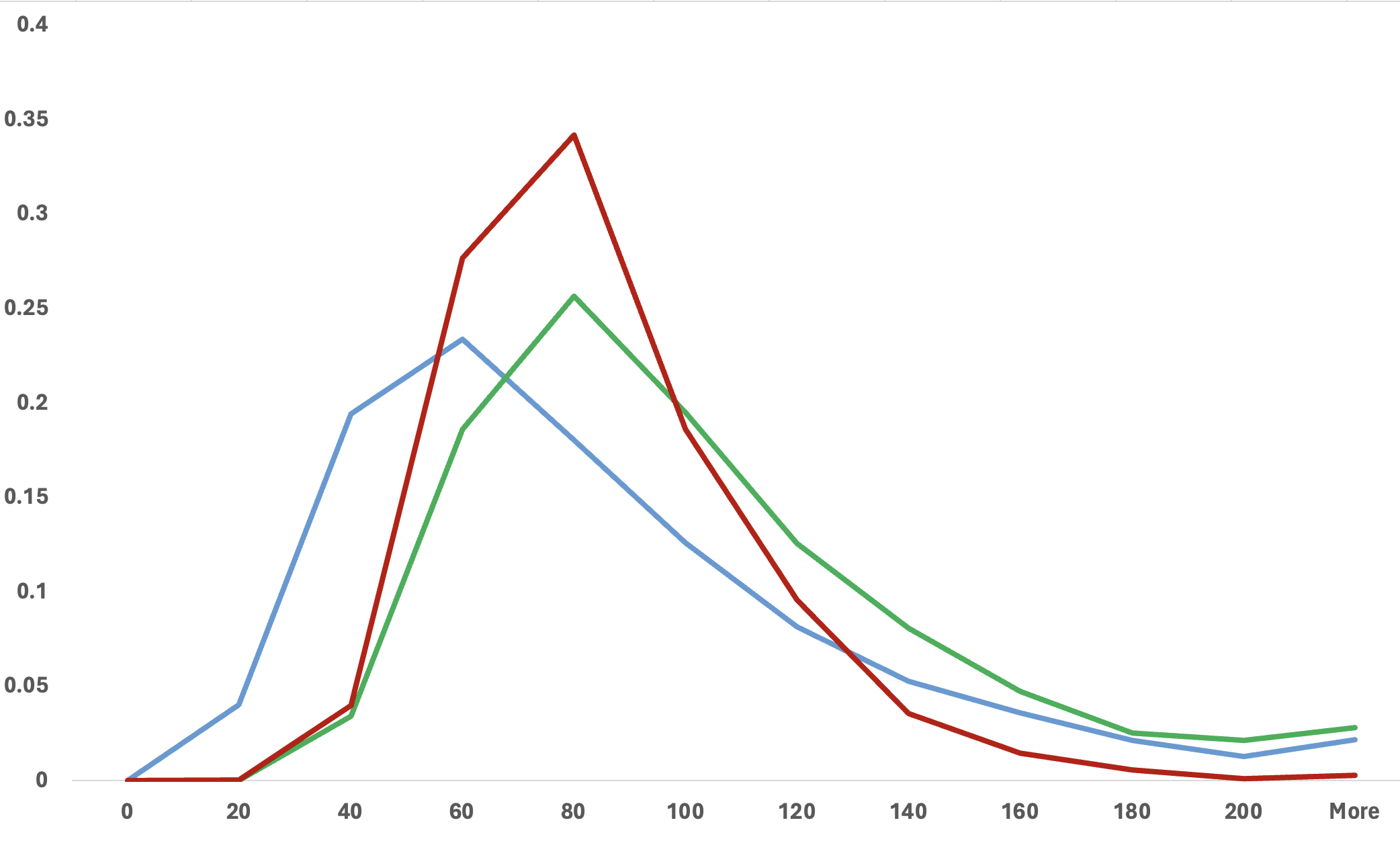


**(C)**

**Cluster Density**

**Cluster Radius (nm)**

**DBSCAN**

**FOCAL^1^**

**FOCAL^PC2^**


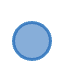

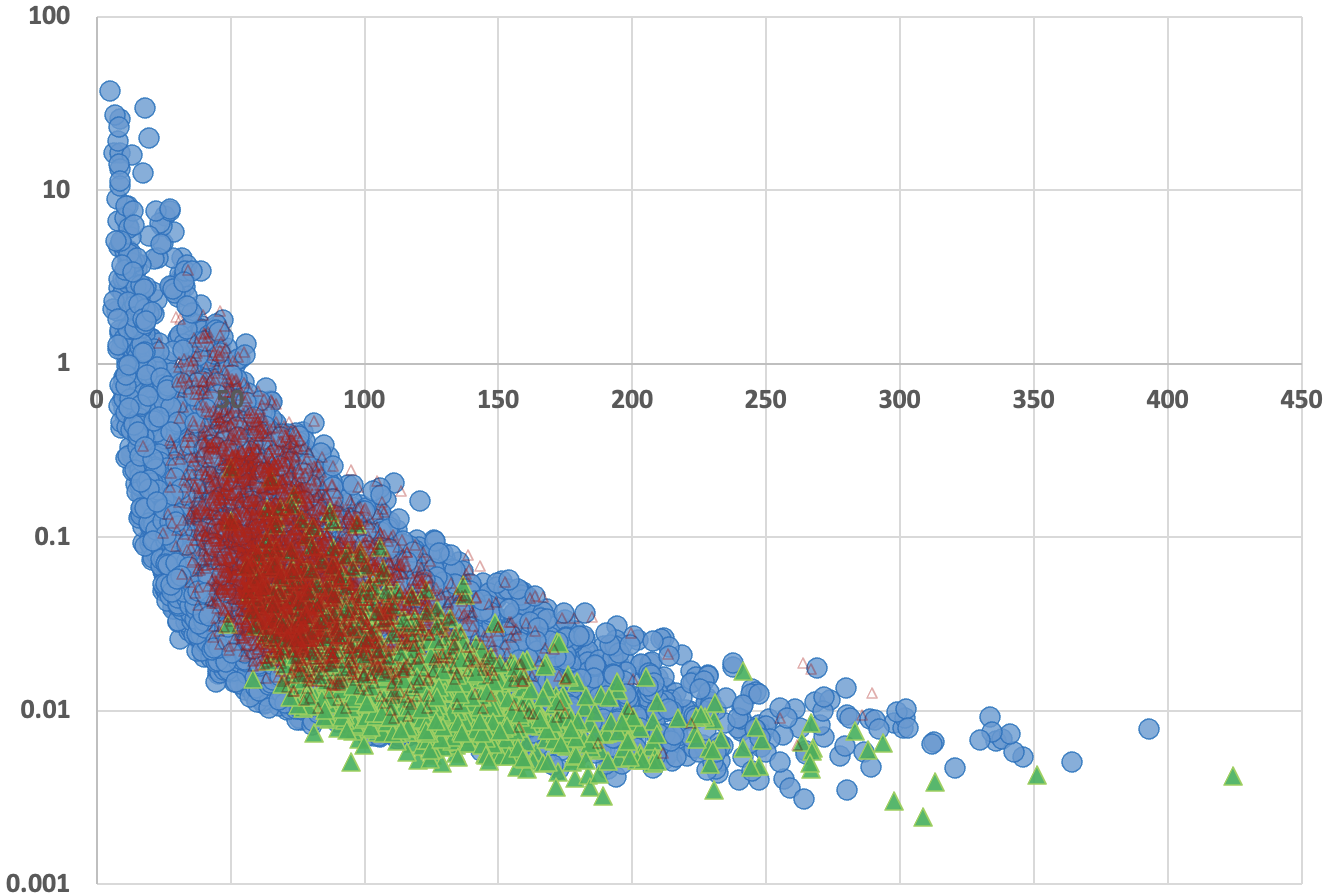

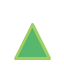

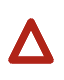


**Probability**

**Cluster Radius (nm)**

**(A)**

**Probability**

**FOCAL^1^**

**FOCAL^PC2^**

**DBSCAN**


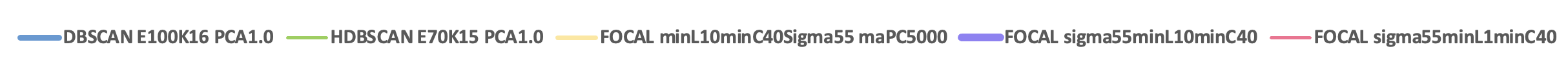

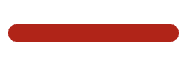

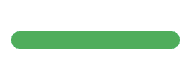


**(B)**

**Cluster Density**


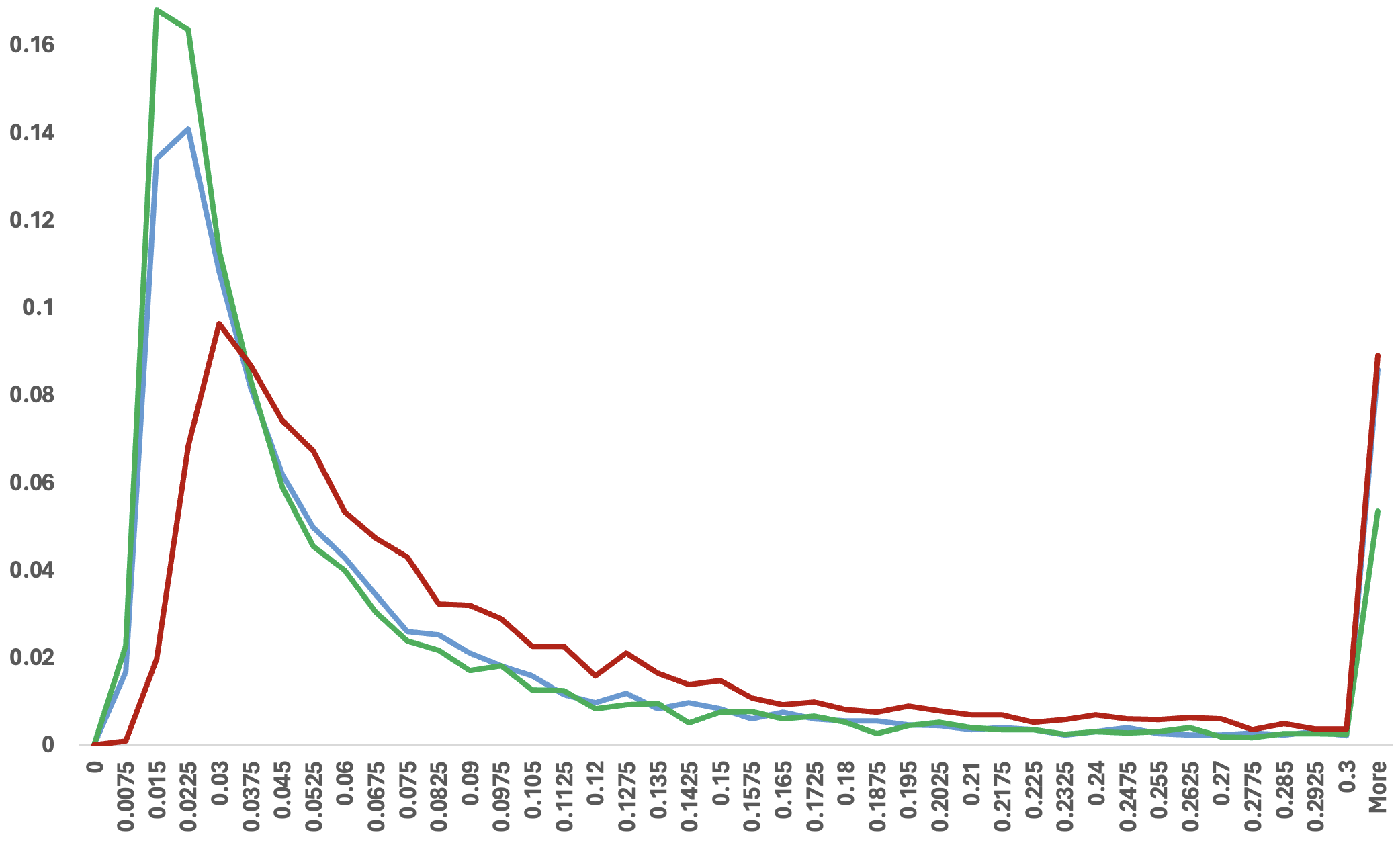


**Supplementary Figure 4. Comparison of PD cluster radius and density as reported by DBSCAN, FOCAL^1^, and FOCAL^PC2^**. The versions of the algorithms tested were DBSCAN with E100, K16 PCA1.0 (DBSCAN), FOCAL Sigma55, minL10, minC40 (FOCAL^1^), and FOCAL Sigma55, minL1, minC40, maPC5000 (FOCAL^PC2^). **A.** Range of cluster radii identified by each algorithm. **B.** Range of cluster densities identified by each algorithm. In the red square is a close-up into the higher range of densities which are not included in the first histogram. **C.** Scatter plot showing the correlation between cluster radius and density in DBSCAN^1^ (blue), FOCAL^1^ (green), and FOCAL^PC2^ (red). The mean and median numbers of clusters per image in DBSCAN^P1^ [162.619, 146.5], in FOCAL^1^ [85.79, 75], and in FOCAL^PC2^ [55.92, 47]. The mean percentage of localizations identified as clustered in DBSCAN^P1^=39.7%, in FOCAL^1^=33.7%, and in FOCAL^PC2^=21.6%.


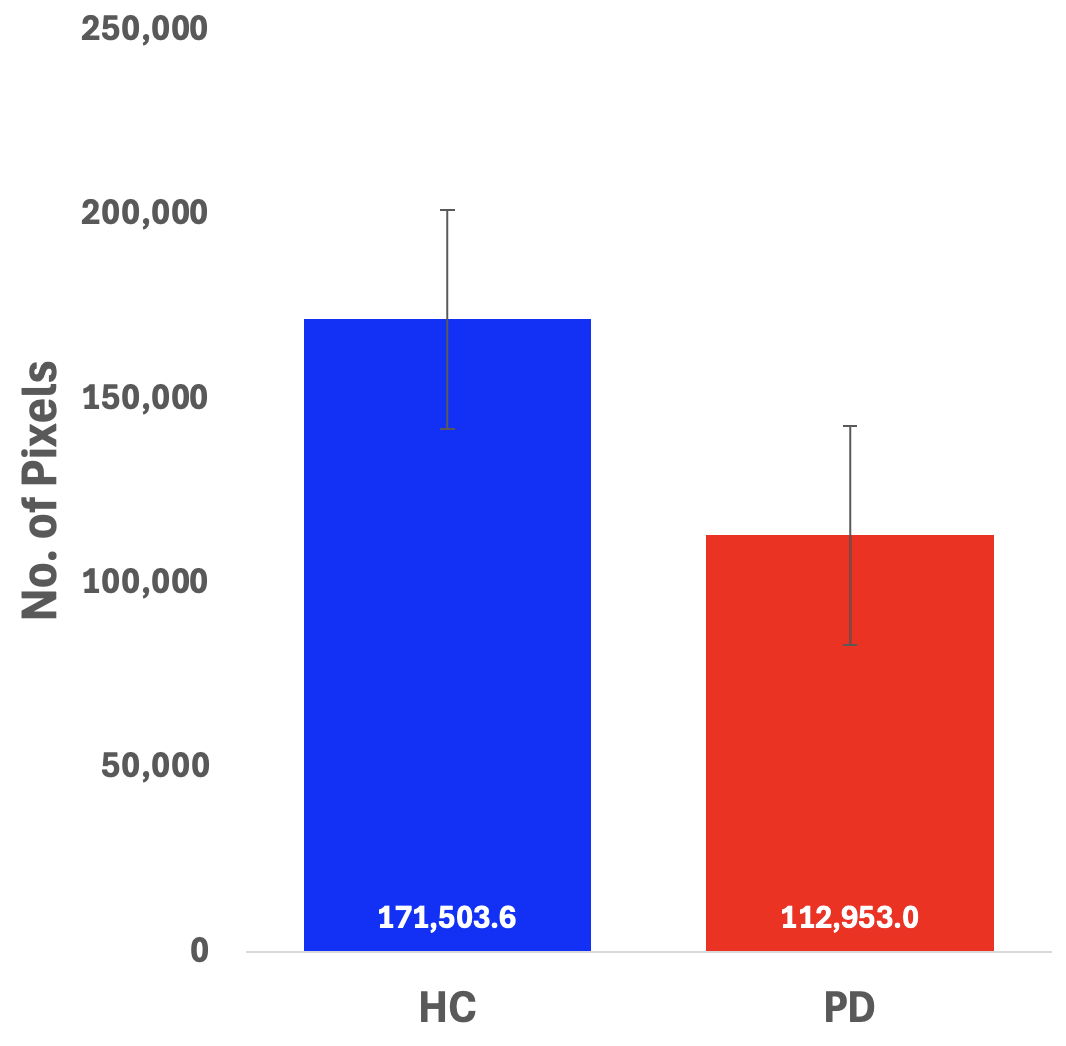


*

**Supplementary Figure 5. Mean number of pixels showing PGP9.5 signal is significantly higher in HC than in PD wide-field images.**  The ROI for each wide-field image was selected manually around the PGP9.5 signal of the gland or the nerve bundle using Fiji’s ImageJ software. A threshold was applied to the image using the adjust-threshold tool and then the number of pixels were summed up using the analyze-histogram tool. Bars represent average pixel number from 18 images for HC and PD. Two-tailed Mann-Whitney U test, * p> 0.05.

**Supplementary Figure 6. No significant correlation between subject’s age and the number of clusters in the subject’s images.** Scatter plot showing number of p-aSyn clusters of each PD patient and HC subjects and their age. No correlation was found between the subject age and the number of p-aSyn clusters detected. The correlation coefficient between the number of clusters and age has a similar and negative slope (-0.25 for HC and -0.34 for PD patients).


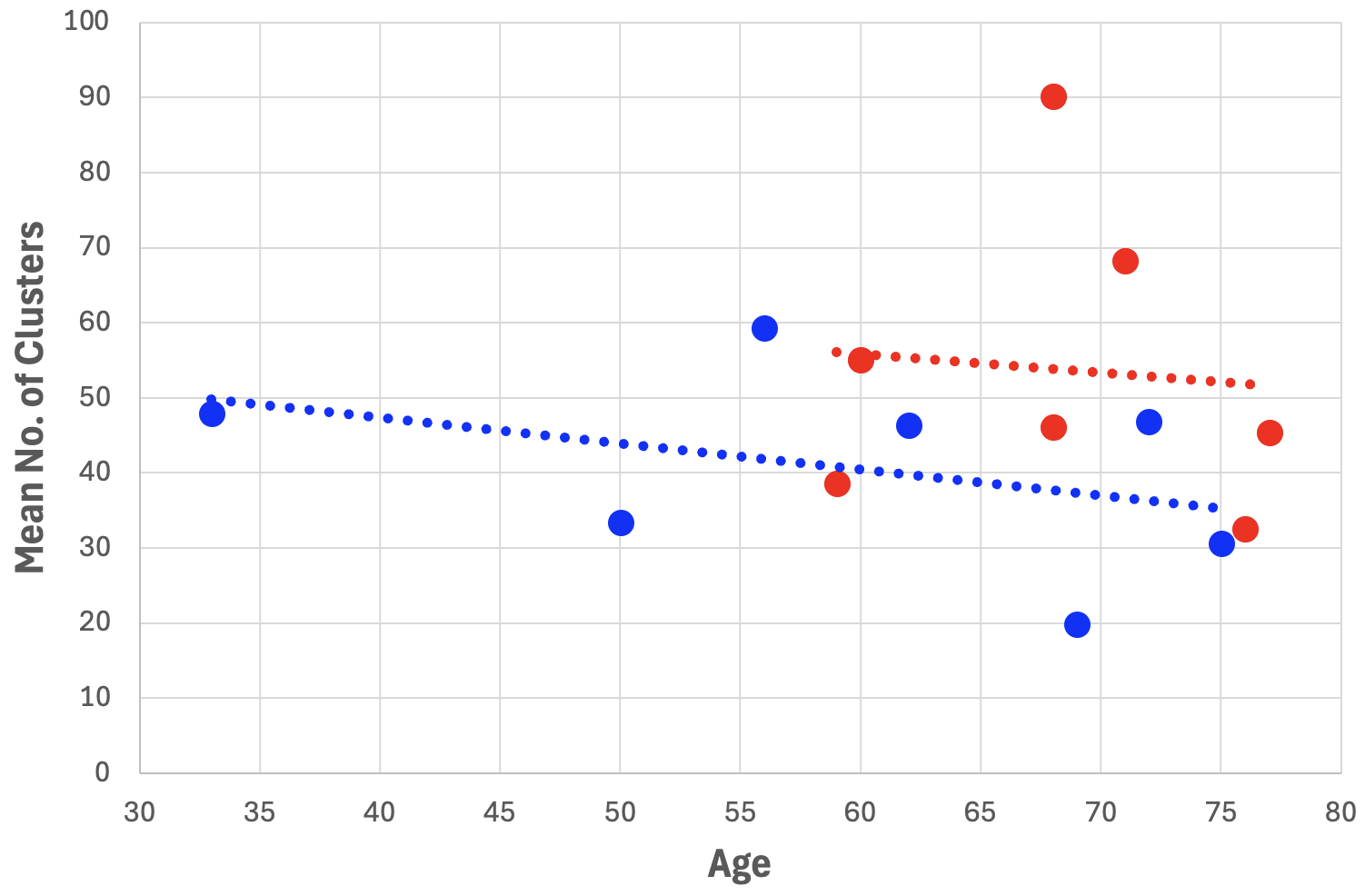

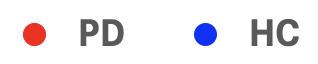

Supplement: Supplementary file 1 [file Table_1.docx]
